# *DMRscaler*: a scale-aware method to identify regions of differential DNA methylation spanning basepair to multi-megabase features

**DOI:** 10.1186/s12859-022-04899-1

**Published:** 2022-09-05

**Authors:** Leroy Bondhus, Angela Wei, Valerie A. Arboleda

**Affiliations:** 1 Department of Human Genetics, David Geffen School of Medicine, UCLA, 615 Charles E. Young Drive South, Los Angeles, CA 90095 USA; 2 Bioinformatics Interdepartmental PhD Program, David Geffen School of Medicine, UCLA, Los Angeles, CA 90095 USA; 3 Department of Pathology and Laboratory Medicine, David Geffen School of Medicine, UCLA, Los Angeles, CA 90095 USA; 4 Department of Computational Medicine, David Geffen School of Medicine, UCLA, Los Angeles, CA 90095 USA; 5 Molecular Biology Institute, UCLA, Los Angeles, CA 90095 USA; 6 Jonsson Comprehensive Cancer Center, UCLA, Los Angeles, CA 90095 USA

**Keywords:** Rare disease, Epigenome, Scale, DNA methylation, Chromatin, Arboleda-Tham syndrome, Sotos syndrome, Weaver syndrome

## Abstract

**Background:**

Pathogenic mutations in genes that control chromatin function have been implicated in rare genetic syndromes. These chromatin modifiers exhibit extraordinary diversity in the scale of the epigenetic changes they affect, from single basepair modifications by DNMT1 to whole genome structural changes by PRM1/2. Patterns of DNA methylation are related to a diverse set of epigenetic features across this full range of epigenetic scale, making DNA methylation valuable for mapping regions of general epigenetic dysregulation. However, existing methods are unable to accurately identify regions of differential methylation across this full range of epigenetic scale directly from DNA methylation data.

**Results:**

To address this, we developed DMRscaler, a novel method that uses an iterative windowing procedure to capture regions of differential DNA methylation (DMRs) ranging in size from single basepairs to whole chromosomes. We benchmarked *DMRscaler* against several DMR callers in simulated and natural data comparing XX and XY peripheral blood samples. *DMRscaler* was the only method that accurately called DMRs ranging in size from 100 bp to 1 Mb (pearson's r = 0.94) and up to 152 Mb on the X-chromosome. We then analyzed methylation data from rare-disease cohorts that harbor chromatin modifier gene mutations in *NSD1*, *EZH2*, and *KAT6A* where *DMRscaler* identified novel DMRs spanning gene clusters involved in development.

**Conclusion:**

Taken together, our results show DMRscaler is uniquely able to capture the size of DMR features across the full range of epigenetic scale and identify novel, co-regulated regions that drive epigenetic dysregulation in human disease.

**Supplementary Information:**

The online version contains supplementary material available at 10.1186/s12859-022-04899-1.

## Background

Genes that regulate chromatin structure and function are critical to coordination of complex developmental trajectories within an embryo. Mutations in these chromatin modifier genes are enriched in clinical cohorts with autism [1–4], congenital heart disease [5, 6] and global developmental delay [3, 5]. Pathogenic mutations in chromatin modifier genes can also result in specific syndromes that have both overlapping and distinct phenotypic features [7–10]. While clinical phenotypes often converge around a common set of chromatin modifier genes, the underlying molecular mechanisms driving these phenotypes are not well characterized.

Chromatin modifiers work in protein complexes to bind chromatin and shape the physical and chemical landscape of the genome, i.e. the epigenome. The regions within the genome where a particular chromatin modifier exerts its influence are critical to defining its role in development. The genomic region controlled by a chromatin modifier can be highly localized, as in methylation of individual cytosine nucleotides which modulates the binding affinity for certain transcription factors (TFs) [11–15], or it can extend across the chromatin landscape more globally, as occurs with the PRM1/2 mediated compaction of the genome during spermatogenesis [16, 17] or *Xist* in condensing the X-chromosome in cells with multiple copies of the X-chromosome [18–20]. Between the local and the global are a diversity of epigenetic features that exist at intermediate scales from tens of kilobases to many megabases. These include features such as polycomb repressive domains (PRDs) [21–23] and topologically associated domains (TADs) [24] and co-regulated gene clusters. These intermediate-sized features coordinate higher order patterning events throughout the genome in development, such as PRD regulation of *Hox* segmentation patterning [25], or organization of olfactory receptor gene clusters into TADs [26] with interdependent epigenetic regulation of the member olfactory receptor genes [27, 28]. A comprehensive understanding of chromatin modifiers requires understanding the scale of their effect on the epigenetic landscape.

While the direction of causality is still an open question for the interaction between many epigenetic features, changes in DNA methylation (DNAme) are often associated with changes in other epigenetic features across the range of epigenetic scale. DNAme is the covalent addition of a methyl group to a single cytosine nucleotide usually in the context of a CpG dinucleotide [29]. While DNAme directly alters the binding affinity for a set of DNA binding proteins [11–15], it is also associated with higher order epigenetic features. At promoters and enhancers DNAme tends to be inversely correlated with gene activity [30, 31]. Over the tens to hundreds of kilobases of PRDs, DNA methylation is depleted by the antagonistic action of the polycomb repressive complex [32, 33], and as a result changes in polycomb activity over PRDs are often associated with differential methylation [33]. Megabase scale domains of active and inactive chromatin can be reliably predicted from DNAme patterns [34], and in colon cancer, changes to DNAme have been reported to overlap with these megabase-sized inactive domains [35].

Phenotypic variability and genetic heterogeneity can make the diagnosis of rare syndromes challenging. Even more challenging is the interpretation of the clinical significance of rare genetic variants identified in whole genome sequencing studies in patients with rare disease. In the absence of clear functional data, these genetic variants are annotated as variants of unknown significance (VUSs). One method to distinguish between pathogenic and benign variants is to identify common patterns of differential DNAme from patients with known pathogenic mutations in the same gene, a methylation signature [9, 10, 36]. The presence of a DNAme signature suggests that common epigenetic marks are associated with pathogenic mutations in specific genes. However, directly linking observed DNAme change to the epigenetic mechanisms contributing to disease remains an open challenge.

Despite the known diversity in scale of differential DNA methylation features, no existing methods are designed to identify regions of differential methylation (DMRs) across the full range of scale from genome-wide methylation data. Instead, existing methods are designed to identify DMRs on the scale of single genes or enhancers, which provides important but incomplete information towards understanding the full epigenetic architecture. This leaves a gap in using DNA methylation to understand the dynamics of co-regulated genes and regions in a broader epigenetic context.

Here we describe a method, *DMRscaler*, that accurately identifies regions of differential methylation that can span several basepairs up to those existing at much larger scales spanning many megabases of sequence across the global DNA methylation landscape. We demonstrate the dynamic range of our differential methylation caller by simulating DMRs varying in size from 100 bp to 1 Mb and testing its performance relative to existing methods. Additionally, we use real methylation data to test for sex differences in DNA methylation where *DMRscaler*, at its highest level calls the X-chromosome as a single differentially methylated feature while still calling small, gene-level DMRs on the autosomes. Finally, we show that pathogenic mutations in chromatin modifier genes are associated with differential methylation of large and highly conserved gene-clusters such as the *HOX* and *PCDH* gene clusters. By bridging the local and the global, *DMRscaler* can provide a broadened view of differential DNA methylation structure.

### Implementation

The primary motivation for DMRscaler is to enable robust and accurate identification of regions of differential methylation that may exist at dramatically different scales. In DNA methylation data, DNA methylation is measured as the proportion of cytosines methylated at a given CpG site in the genome across all cells in a sample. This proportion is the β (beta) value of that site, with β = 0 being completely unmethylated and β = 1 being completely methylated. The distribution of β values for all CpGs across the genome follows a bimodal distribution (Additional file 1: Figure S1). DMRscaler takes as input a set of CpG probes with their chromosome, genomic position, and pre-computed p-value for individual CpG level significance, and individual CpG level p-value cutoff threshold for the desired Type I error control level. Estimation of the p-value cutoff for desired Type I error control should be done at the level of individual CpG level to avoid identifying DMRs that represent correlated blocks of CpGs that are not associated with the condition of interest. One method for estimation of this cutoff value, implemented with the *DMRscaler* package, is to repeat the individual CpG level significance testing with permutations of the case and control labels and compare the distributions of CpG significance values from these random permutations against the true case–control partition, however other methods could also be used. By working on an input of p-values, DMRscaler gives the user the flexibility to choose the statistical test that is most appropriate for their experimental design.

**Fig. 1:**
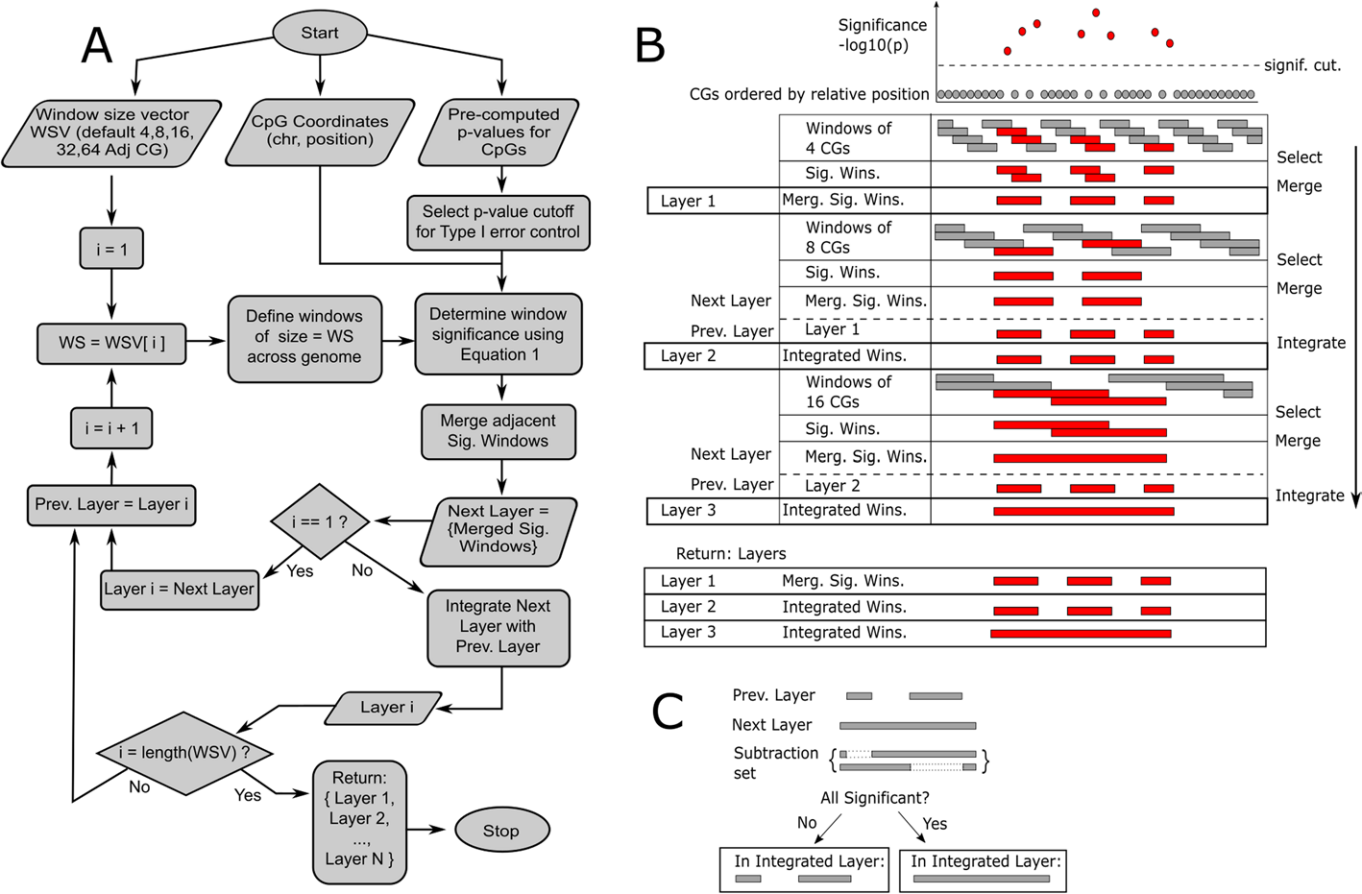
Outline of *DMRscaler* method. **A** Flowchart of decision tree for *DMRscaler*. Starts with specification of a window size vector that lists the window sizes to use for identifying DMRs in each layer, and the coordinates of CpG probes with precomputed p-values for each CpG. Additionally Type I error control should be done using these precomputed p-values by setting a p-value cutoff value to ensure that identified DMRs have the expected Type I error rate with respect to their association with the condition of interest. Window significance is determined using Eq. (1). Adjacent significant windows are merged forming the *Next_Layer*. For the first iteration, the returned *Layer_1* is set to this *Next_Layer*, for subsequent iterations the returned *Layer_i* is set to the result of integration of *Next_Layer* with *Prev_Layer*. Integration of layers is described in 1C. *Prev_Layer* is updated to *Layer_i* before proceeding to iteration i + 1. After the largest window size layer is generated, a list is returned of the results from each iteration of the algorithm. **B** Graphical description of algorithm. At the top shows representation of CpGs ordered by position and associated with a significance value. Windows are laid over the ordered CpGs and selected if the window score is significant. Adjacent windows are then merged. If a *Prev_layer* has been assigned, then integration occurs. **C** Integration procedure. For each *Next_Layer* DMR, all overlapping *Prev_Layer* DMRs are identified. A subtraction set is generated by individually subtracting each overlapping *Prev_Layer* DMR from the *Next_Layer* DMR. Subtraction involves removing overlapping CpGs from the *Next_Layer*. If all elements of the subtraction set are significant when rescored with the window scoring function, then the *Prev_Layer* and *Next_Layer* regions are merged in the *Integrated_Layer*, otherwise the *Prev_Layer* DMRs are used in the *Integrated_Layer*. This procedure ensures that the broader *Next_Layer* DMRs are only included if no single *Prev_Layer* DMR was responsible for the significance of the region identified in the *Next_Layer*

To identify regions that are characterized by differentially methylated CpGs (i.e. differentially methylated regions or DMRs), DMRscaler uses a sliding window scheme (Fig. 1A,B). Windows are defined by a count of adjacent CpGs rather than by the span of the genomic region. The use of a count of adjacent CpGs for window definition makes *DMRscaler* agnostic to CpG density. This allows *DMRscaler* to scan regions with low CpG coverage, such as heterochromatin, that might be missed using a distance parameter between CpGs. Future iterations of the method may allow specification of fixed genomic interval widths for defining DMRs. However, a limitation here is that it is subject to the choice of methylation sites included on the currency DNA methylation chips.

Region-wide significance is taken to be the probability of obtaining within a window the set of CpG ranks or a set of more extreme ranks by random chance, given as a prior that the most significant CpG in the window has already been drawn. The null hypothesis then is that the ranks of the CpGs within a window are equally or less extreme than would be expected by a random draw from the complete set of CpG ranks given as a prior that the most significant CpG in the window has been drawn. The product of a sequence of hypergeometric tests is used to determine the region-wide significance of each window as described by the function

1 $$p_{region} = \prod\limits_{i = 1}^{m} h yper_{CDF} (k_{i} ,n_{i} ,N_{i} ,K_{i} )$$

where CpGs in the window, after excluding the most significant overlapping CpG from the window, are ordered from least to most significant going from $i = 1$ to $i = m$. Variables are defined as follows:

$$m = {\text{total}}\;\# \;{\text{CpGs}}\;{\text{in}}\;{\text{the}}\;{\text{window}}$$

$$k_{i} = \# \;{\text{of}}\;{\text{CpGs}}\;{\text{in}}\;{\text{window}}\;{\text{with}}\;{\text{rank}}\;{\text{greater}}\;{\text{than}}\;{\text{or}}\;{\text{equal}}\;{\text{to}}\;{\text{K}}_{{\text{i}}}$$

$$n_{i} = \left\{ {\begin{array}{*{20}l} m \hfill & {{\text{if }}i = 1} \hfill \\ {k_{i - 1} - 1} \hfill & {{\text{otherwise}}} \hfill \\ \end{array} } \right.$$

$$N_{i} = \left\{ {\begin{array}{*{20}l} {{\text{total}}\;\# \;{\text{of}}\;{\text{CpGs}}\;{\text{in}}\;{\text{dataset}}} \hfill & {{\text{if }}i = 1} \hfill \\ {K_{i - 1} - 1} \hfill & {{\text{otherwise}}} \hfill \\ \end{array} } \right.$$

$$K_{i} = {\text{rank}}\;{\text{of}}\;i{\text{th}}\;{\text{CpG}}$$

The hypergeometric cumulative distribution function, $hyper_{CDF}$, is set up to return the likelihood of obtaining $k_{i}$ or more successes in $n_{i}$ draws from a population of size $N_{i}$ where there are $K_{i}$ success cases total. At each step from $i = 1$ to $i = m$, the function determines the probability of having $k_{i}$ or more CpGs of rank $K_{i}$ or higher from $n_{i}$ random draws in a population of $N_{i}$ CpGs. The variables update at each step to account for how the likelihood has changed given the information contained in the previous step. The updates to the variables make the result of each hypergeometric test independent. The product of these independent tests then gives the region-wide significance value that effectively represents the probability of associating CpGs with the ranks observed, or ranks more extreme, by random chance in a window of the given size. This in effect then defines DMRs as regions that have a statistically significant association by adjacency of individually significant, by FDR or FWER control, CpGs. It should be noted that this procedure means that while region significance is linked to individual CpG level significance, these two metrics of significance are distinct. For example, if a region's most significant individual level CpG significance value is *p* = 0.01, but the region wide significance value is *p* = 1e − 12, then the region is almost certainly consisting of CpGs that are truly associated by adjacency (e.g. regulated in some respect as a unit relative to the set of all measured CpGs), however the determination of whether the differential methylation of this region is truly associated with the biological condition of interest should be based on individual CpG level significance.

Since there are nearly as many windows that can be tested for significance as there are CpGs included in the dataset, multiple testing must be accounted for to avoid excessive Type I errors. To do this, DMRscaler gives options to use Bonferroni correction procedure to control the family-wise error rate, or the Benjamini-Yekutieli procedure [37] to control the false discovery rate. With either of these the user supplies a region-wide significance threshold below which regions are considered significant. Both procedures are implemented so that the number of tests performed is equal to the number of measured probes below the user specified individual level CpG p-value cutoff. We observed conservative FDR control in simulations varying both the individual CpG level FDR threshold and the region-wide significance threshold (Additional file 1: Fig. S2).

In order to define DMRs that can vary dramatically in scale within the same analysis, we implemented the sliding window procedure iteratively increasing the size of the windows used to identify DMRs at each step of the iteration. The set of DMRs called at each step of the iteration is defined as a *layer* of the procedure, with layers named by the size of windows used for calling DMRs within that layer and indexed by the iteration step number. For example, if windows of 4 adjacent CpGs are used first to call DMRs, then *layer_1* (or layer_1_) and the *4_adjacent_CpG_layer* are synonymous. An important step to accurately identify the scale of DMRs and avoid overinflation of DMR size is the inclusion of a step to integrate the results across these layers (Fig. 1B, C). This integration procedure works as the method iterates from one layer to the next by testing whether a tentatively significant window called at the current layer remains statistically significant after the removal of CpGs from each overlapping DMR from the previous layer individually. For example, if a given window at the current layer has 100 CpGs and is considered tentatively significant, and there are 2 overlapping DMR from the previous layer with 20 CpGs and 30 CpGs, the DMR at the current layer is only retained if the 80 CpGs left after removal of the 20 CpG DMR from the first overlapping previous layer DMR are still considered significant as a region AND the 70 CpGs left after the removal of the 30 CpG DMR from the second overlapping previous layer DMR are still considered significant as a region. Otherwise, if either of these remaining 80 CpGs or 70 CpGs are not significant, then the current layer does not consider the 100 CpGs to be a DMR and instead the current layer is set to include the 20 CpG and 30 CpG DMRs from the previous layer, thereby propagating these DMRs from the previous layer.

It should be noted that no additional multiple testing correction is carried out to account for the number of layers used for identifying DMRs. As each layer is dependent on the same base layer of individual CpGs for estimation of significance, tests across layers are not independent and so our intuition is that it is reasonable to perform FDR or FWER correction only within layers. More rigorous statistical accounting for the dependency structure of this layer integration procedure is a challenge we have left as a future direction of study.

Here will elaborate on this procedure a little more formally. To begin, the smallest window size is used as a parameter to identify DMRs and build *layer*_*1*_ of the output. As a note, the term 'layer' is used to describe the resulting set of DMRs constructed with a given window size parameter to suggest the relation of the results of each iteration of the algorithm. The construction of each successive layer either expands, adds, or retains DMRs from the *previous layer*, and so there is a hierarchical relation between DMRs across layers. DMRs in lower layers will always map to some DMR in upper layers, ensuring that *layer*_*i*_ will always be a subset of *layer*_*i*+*1*_. To define the *next layer*, the following steps are repeated for windows of the next largest size: overlaying windows, identifying windows significantly enriched in differentially methylated CpGs, and merging significant windows (Fig. 1A, B). From the second layer onward, an additional step to integrate the results from the *previous layer* is performed. This is achieved by subtracting each *previous layer* DMR individually from any overlapping tentative DMR in the *next layer*, and retesting all generated reduced DMR sets in the *next layer* for significance (Fig. 1C). If a tentative DMR in the *next layer* remains significant following the subtraction of each overlapping *previous layer* DMR individually, then the tentative *next layer* DMR is retained in the new *integrated layer*. Otherwise, the overlapping *previous layer* DMRs are retained without change, replacing the tentative *next layer* DMR in the *integrated layer*. Additionally, any *previous layer* DMRs that did not overlap a DMR in the *next layer* are added to the *integrated layer*. In this way, at each iteration of the algorithm DMRs are either added, expanded, or consolidated from the *previous layer* to the *integrated layer* but never lost. As the algorithm proceeds, the *previous layer* is updated to the most recent *integrated layer* before the next step of integration is done with the *next layer*. Through iteratively calling DMRs using windows of increasing size and integrating the results, *DMRscaler* is able to identify DMRs that vary dramatically in terms of scale.

## Methods

### Cell culture

For Arboleda-Tham Syndrome data, fibroblast cell lines were derived from skin punch biopsies performed on the proband and one or both unaffected parents. This project was approved by the UCLA Institutional Review Board #11-001087. All individual level-data was de-identified prior to analysis. Fibroblast cell culture lines were created through the UCLA Pathology Research Portal and fibroblast cell lines were established and grown in DMEM (Gibco™), 10% FBS (Heat-inactivated Fetal Bovine Serum, Thermofisher), 1% Non-essential Amino Acid (Gibco™) and 1% PenStrep at 37 °C in 5% CO_2_ incubators. Cell lines were tested for mycoplasma on a monthly basis.

### DNA methylation studies

For Arboleda-Tham Syndrome methylation studies, DNA was extracted from patient-derived fibroblast cell lines. The specific mutation for each line is given in Additional file 1: Table S1. DNA samples were bisulfite converted and run on the Illumina MethylationEPIC Array (850 k EPIC array) as previously described [38] at the UCLA Neuroscience Genomics Core to generate idat files. QC on the resulting idat files was done using the MINFI package, and probes overlapping SNPs were removed [39]. After QC, 852,671 of 865,919 measured CpGs remiained, after removal of sex chromosome CpGs, 832,159 measured CpGs remained. Preprocessing and normalization of individual probes was done using background correction [40] and functional normalization [41].

### Data sources

Publically available datasets of peripheral blood methylation data for control, Weaver Syndrome and Sotos Syndrome patients were downloaded from the Gene Expression Omnibus (GEO) resource [42, 43] with accession number GSE74432 [10].

### Simulation

**Fig. 2:**
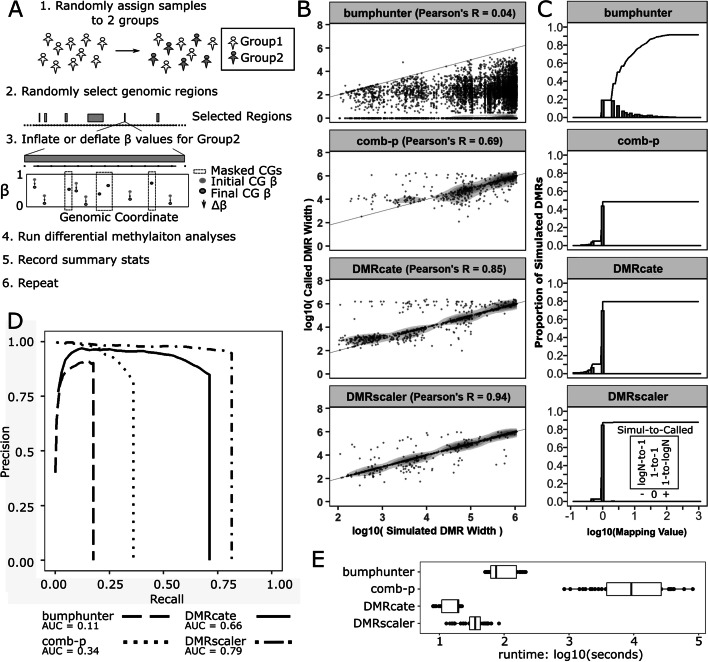
Simulation of DMRs ranging in size between 1 kb and 1 Mb for comparison of methods. **A** Graphical description of simulation design. First, samples are randomly assigned to one of two groups. Second, non-overlapping regions of the genome are randomly selected to be DMRs. Third, over selected DMRs one group has the β value of non-masked CpGs inflated or deflated by Δβ. Next all differential methylation methods are run and relevant summary statistics are recorded. This procedure is repeated a number of times to generate additional data points. **B** Simulated DMR Widths v Called DMR Widths plotted on log10 scale. Pairs are formed between simulated and called DMRs if there is any overlap between the two. **C** Mapping Values plots. The mapping value is calculated for each simulated DMR and is either the inverse of the number of simulated DMRs sharing an overlapping called DMR or else it is the number of called DMRs overlapping the given simulated DMR, whichever is more extreme. Log values > 0 imply multiple DMRs called per simulated DMR. Value < 0 imply multiple simulated DMRs overlap single called DMR. Value = 0 implies one DMR called per DMR simulated. The plotted line indicates the cumulative proportion of simulated DMRs up to the given mapping value. **D** Feature level Precision-Recall Curves for each method, see methods for details on calculation. **E** Time for each method to run on the simulated dataset for each parameter set combination

To demonstrate how *DMRscaler* distinguishes itself from other methods, we simulated differentially methylated regions (DMRs) ranging in size across several orders of magnitude (Fig. 2A).

DNA methylation measured on the Infinium HumanMethylation450 BeadChip (450 K array) from whole blood for 53 controls from GEO (GSE74432) was used as a foundation for the simulation [10]. Real data was used as the foundation in order to capture the natural biological and technical variability present in DNA methylation array data. QC on the resulting idat files was done using the MINFI package, and probes overlapping SNPs were removed [39]. After QC, 468,162 of 485,512 measured CpGs remained, and after removal of the sex chromosomes 456,514 measured CpGs remained. Preprocessing and normalization of individual probes was done using background correction [40] and functional normalization [41].

Regions for artificially introducing DMRs were selected at random across the genome but subject to the following constraints. DMRs specified as 0.1–1 kb in size were required to have at least 3 CpGs represented on the 450 K array (CpGs), those 1–10 kb in size were required to have at least 6 CpGs, those 10–100 kb in size were required to have at least 9 CpGs, those 0.1–1 Mb in size were required to have at least 12 CpGs. Additionally, to avoid miscounting, DMRs were introduced such that they were spaced at least 10 CpGs apart from any other introduced DMR. Distribution of CpG counts in simulated DMRs against simulated DMR sizes are shown in Additional file 1: Fig. S3.

All 450 k array samples used were from control whole blood DNA, so for each run of the simulation samples were pulled at random into one of two groups, Group1 and Group2. Each group consisted of 8 samples drawn without replacement from the pool of 53 samples.

Before artificially introducing the DMRs to the real data matrix, a proportion of CpGs within each DMR, excluding the first and last CpGs, specified by the *noise* parameter were randomly masked and kept at their original β values. This was done to model the variability of methylation state of neighboring CpGs in real data. Values for the *noise* parameter tested were 0, 0.25 and 0.5, corresponding to 0%, 25%, and 50% of CpGs overlapping a simulated DMR being masked. Then, the mean β value of CpGs within each DMR was measured for Group1 and Group2. The β values of the group with the greater mean β value would have all non-masked CpGs inflated by an amount specified by the *Δβ* parameter. Simulations were run with *Δβ* values of 0.1, 0.2, and 0.4 to model small, modest, and large effect DMRs respectively. If this resulted in any samples having a β value greater than 1, the β values for that CpG were divided by the max β value for that CpG to bring values back to the range of 0–1.

Following the introduction of artificial DMRs into the dataset, *DMRscaler*, *bumphunter *[44]*, comb-p *[45] and *DMRcate *[46] were run on the dataset and the results tabulated. *DMRscaler* was tested using a window_size_vector of c(4, 8, 16, 32, 64) adjacent CpGs, locs_pval_cutoff corresponding to the individual level CpG *p*-value at which FDR < 10% is achieved, region_signif_cutoff = 0.01 corresponding to the region level significance threshold for calling a region as a DMR after multiple testing correction, and region_signif_method = "benjamini-yekutieli" specifying the benjamini-yekutieli procedure as the method for Type I error control. *Bumphunter* was tested with MaxGap = 1e6 with loess smoothing enabled. *Comb-p* was tested with dist = 1e6, step = 5000, seed = 1e-3, region-filter-p = 0.1 (Additional file 1: Fig. S4). *DMRcate* was tested with lambda = 1e6, C = 2000. Parameter sets for methods were chosen to facilitate identification of larger DMRs for output more comparable to *DMRscaler*.

To benchmark each method’s performance several metrics were used including proportion of CpGs in DMRs that are differentially methylated, precision, recall, specificity, F1, Matthew's correlation coefficient (MCC), and the area under the precision recall curve (AUCPR). These metrics were recorded for analysis at the feature, basepair, and CpG probe level, where feature level assessment treated each simulated DMR as a single positive feature, the basepair level treated each basepair overlapping a simulated DMR as a positive feature, and the CpG probe level treated each CpG probe overlapping a simulated DMR as a positive feature. The basepair and CpG level assessments are based on direct counts of true and false positives and negatives. The feature level assessment was conducted following the framing of the problem for measuring precision and recall for time series proposed by Tatbul and colleagues, which is generally appropriate for other forms of range data when identification of individual features is of interest [47]. Each simulated DMR was considered as a true feature with true positive (TP) and false negative (FN) attributes. Each called DMR was considered a called feature with TP and false positive (FP) attributes. Called DMRs were ordered by their p-value for all methods. The precision-recall curve was generated by measuring precision and recall with stepwise inclusion of the next highest scoring or most significant called DMR. At the n-th step precision and recall were measured as

2 $$Precision = \frac{{TP_{Called\_DMRs} }}{{TP_{Called\_DMRs} + FP_{Called\_DMRs} }}$$

2.1 $$TP_{Called\_DMRs} = \frac{{\sum\limits_{i = 1}^{n} {TP_{{Called\_DMR_{i} }} } }}{n}$$

2.2 $$FP_{Called\_DMRs} = \frac{{\sum\limits_{i = 1}^{n} {FP_{{Called\_DMR_{i} }} } }}{n}$$

3 $$Recall = \frac{{TP_{Simulated\_DMRs} }}{{TP_{Simulated\_DMRs} + FN_{Simulated\_DMRs} }}$$

3.1 $$TP_{Simulated\_DMRs} = \frac{{\sum\limits_{j = 1}^{m} {TP_{{Simulated\_DMR_{j} }} } }}{m}$$

3.2 $$FN_{Simulated\_DMRs} = \frac{{\sum\limits_{j = 1}^{m} {FN_{{Simulated\_DMR_{j} }} } }}{m}$$

where $TP_{{Called\_DMR_{i} }}$ is the proportion of the i-th called DMR overlapping simulated DMR regions, $FP_{{Called\_DMR_{i} }}$ is the proportion of the i-th called DMR not overlapping a simulated DMR region, $TP_{{Simulated\_DMR_{j} }}$ is the proportion of the j-th simulated DMR overlapping any of the 1 to n-th Called DMRs, $FN_{{Simulated\_DMR_{j} }}$ is the proportion of the j-th simulated DMR overlapping any of the 1 to n-th Called DMRs, $n$ is the number of most significant called DMRs used at the n-th step, and $m$ is the total number of simulated DMRs. The feature level measure of precision and recall gives equal weight to each simulated DMR so that large simulated DMRs do not dominate the signal.

In addition to precision and recall, several other metrics that were included in a recent benchmark of DMR callers by Mallik et al. [48] were used to assess method performance in the simulation. All of these metrics were measured on the set of called DMRs that had an adjusted region-wide significance *p* < 0.01 for each method. Specificity is a measure of the true negative rate, and is measured as the proportion of true negatives as a fraction of total negative features, and is calculated by the equation:

4 $$Specificity = \frac{{1 - FN_{Simulated\_DMRs} }}{N}$$

False Discovery Rate as the inverse of precision gives expected proportion of false results and is given by:

5 $$FDR = 1 - Precision$$

F1 is a measure of a test's accuracy and is given by:

6 $$F1 = 2*\frac{(Precision*Recall)}{{Precision + Recall}}$$

F1 ranges from 0 for worst accuracy to 1 for perfect classification. Finally, the Matthew's correlation coefficient, which is a measure of correlation between predicted and true class labels and is given by:

7 $$MCC = \frac{{\sqrt {Recall*Specificity*Precision*\frac{{TN_{{Called_{DMRs} }} }}{{N_{{Called_{DMRs} }} }}} }}{{\sqrt {(1 - Recall)*(1 - Specificity)*(1 - Precision)*(1 - \frac{{TN_{{Called_{DMRs} }} }}{{N_{{Called_{DMRs} }} }})} }}$$

MCC values at + 1 indicate perfect classification, 0 indicates equivalence with random classification, and − 1 indicates perfect misclassification.

### Rare disease data analyses

For each real data analysis, *DMRscaler*, *bumphunter, comb-p*, and *DMRcate* were used to call DMRs. *DMRscaler* was tested using a window_size_vector of c(4, 8, 16, 32, 64) adjacent CpGs, locs_pval_cutoff corresponding to the individual level CpG p-value at which FDR < 10% is achieved, region_signif_cutoff = 0.01 corresponding to the region level significance threshold for calling a region as a DMR after multiple testing correction, and region_signif_method = "benjamini-yekutieli" specifying the benjamini-yekutieli procedure as the method for Type I error control. *DMRcate* was tested with default parameters, as well as with lambda = 1e6, C = 2000 to capture larger DMRs for output more comparable to *DMRscaler*. *Bumphunter* was tested with default parameters, as well as with MaxGap = 1e6 with loess smoothing enabled.

For the sex analysis, DNA methylation measured on the Infinium HumanMethylation450 BeadChip (450 K array) from whole blood for 53 controls from GEO (GSE74432) with 29 female and 24 male samples was used [10]. QC on the raw idat files was done using the MINFI package, and probes overlapping SNPs were removed [39]. After QC, 468,162 of 485,512 measured CpGs remained. Preprocessing and normalization of individual probes was done using background correction [40] and functional normalization [41]. Individual level differential CpG significance between female and male samples was measured using the Wilcox test to serve as input for *DMRscaler* and *comb-p*. Raw output from each method is provided in Additional file 2: Table S2.

For Arboleda-Tham Syndrome sample analysis, DNA methylation was measured on the Illumina MethylationEPIC Array (850 k EPIC array) with 8 cases and 12 controls. QC on the resulting idat files was done using the MINFI package, and probes overlapping SNPs were removed [39]. After QC, 852,671 of 865,919 measured CpGs remained, after removal of sex chromosome CpGs 832,159 measured CpGs remained. Preprocessing and normalization of individual probes was done using background correction [40] and functional normalization [41]. Individual level differential CpG significance between female and male samples was measured using the Wilcox test to serve as input for *DMRscaler* and *comb-p*. Raw output from each method is provided in Additional file 3: Table S3.

For Weaver analysis, DNA methylation measured on the Infinium HumanMethylation450 BeadChip (450 K array) from whole blood for 8 patients with *EZH2* mutations and 53 controls from GEO (GSE74432) was used [10]. This data comes from a study that found an epigenetic signature specific to Sotos syndrome from *NSD1* mutations using Weaver syndrome samples as a negative control for their classifier [10]. More recently, this data has been used to identify an epigenetic signature specific to Weaver syndrome [9]. QC on the raw idat files was done using the MINFI package, and probes overlapping SNPs were removed [39]. After QC, 468,162 of 485,512 measured CpGs remained, and after removal of the sex chromosomes 456,514 measured CpGs remiained. Preprocessing and normalization of individual probes was done using background correction [40] and functional normalization [41]. Individual level differential CpG significance between female and male samples was measured using the Wilcox test to serve as input for *DMRscaler* and *comb-p*. Raw output from each method is provided in Additional file 4: Table S4.

For Sotos syndrome analysis, DNA methylation measured on the Infinium HumanMethylation450 BeadChip (450 K array) from whole blood for 38 patients with *NSD1* mutations and 53 controls from GEO (GSE74432) was used [10]. QC on the raw idat files was done using the MINFI package, and probes overlapping SNPs were removed [39]. This comes from the same study as the Weaver syndrome data [10]. After QC, 468,162 of 485,512 measured CpGs remained, and after removal of the sex chromosomes 456,514 measured CpGs remiained. Preprocessing and normalization of individual probes was done using background correction [40] and functional normalization [41]. Individual level differential CpG significance between female and male samples was measured using the Wilcox test to serve as input for *DMRscaler* and *comb-p*. Raw output from each method is provided in Additional file 5: Table S5.

### Syndrome DMR overlap analysis

To test for overlapping regions of differential methylation between Arboleda-Tham, Sotos, and Weaver syndrome, the number of measured CpGs considered for DMR detection was downsampled to include only those CpGs measured on both the Infinium HumanMethylation450 BeadChip (450 K array) and the Illumina MethylationEPIC Array (850 k EPIC array). This left 425,733 measured CpGs for calling DMRs.

Overlaps between DMRs were counted between syndromes as was the overlap of gene sets. Gene set overlaps were considered separately to identify genes that may be commonly differentially methylated but identified by non-overlapping regions of the gene, something the direct DMR overlap measure would miss. Raw output of region level and gene level overlaps are provided in Additional file 6: Table S6.

To test whether CpGs identified as belonging to DMRs are enriched between syndromes, that is whether membership of a CpG to a DMR in one syndrome makes it more or less likely to also belong to a DMR in another syndrome, we computed the odds ratios (OR). The OR was calculated by forming a 2 × 2 contingency table with counts of CpGs belonging to DMRs in both syndromes, CpGs belonging to one and not the other, and CpGs belonging to DMRs in neither. The raw counts used in the 2 × 2 contingency tables are given in Additional file 1: Table S7. Odds ratios for all pairs of syndromes are given in Additional file 1: Table S8.

## Results

### *DMRscaler* overview

Our goal in developing *DMRscaler* was to have a method capable of accurately identifying regions that demonstrate differential methylation across the full range of epigenetic scale, from small-promoter to whole-chromosome scale features. The major bottleneck to this goal is that regions of differential methylation show significant variability in methylation state between neighboring CpGs. For example, nearly 20% of neighboring CpGs between 0.5 and 1.0 kb away have a difference in the proportion of methylation greater than 50% (Additional file 1: Figs. S5, S6). When trying to identify DMRs that may span larger genomic regions, such as gene clusters, this variability makes the trivial method of taking contiguous blocks of significant CpGs as the DMRs ineffective. One approach to resolve this issue of high variability is to smooth differential methylation sites based on significance across adjacent CpGs or over some specified genomic interval. However, the smoothing approach is sensitive to the choice of bandwidth parameter used for the smoothing window. Windows that are too small will fail to connect features over larger gaps, windows that are too large will result in excessively broad DMRs. Smoothing alone is therefore inappropriate when features are expected to vary dramatically in terms of scale. To capture potentially noisy features that may vary in size by several orders of magnitude, from the basepair to multi-megabase scale, we need a method that is both robust to noise and that can accurately determine the feature’s size.

To address these limitations in determining the size of a DMR, *DMRscaler* uses an iterative sliding window over the genome (Fig. 1A, B), represented as a partially-ordered set of measured CpGs, and implements an integration step between each iteration of the sliding window (Fig. 1C). The windows at each step identify the set of regions that are enriched in CpGs with significantly different methylation values between cases and controls. By binning CpGs into windows and testing these windows for enrichment in significant CpGs Eq. (1), the algorithm is robust to noise caused by variability in methylation of neighboring CpGs. To address the bias in feature size introduced by preselecting a window size parameter, *DMRscaler* calls significant windows iteratively with a variable increasing size parameter and integrates the result of each iteration with the results from the previous iterations. The integration step (Fig. 1C) is used between the previous (lower) layer, built from smaller windows, and the current (upper) layer to determine which features in the upper layer are already adequately represented by lower layer features and which upper layer features capture a statistically significant association missed by the lower layer features. If an upper layer feature captures a statistically significant association missed in the lower layer then that upper layer feature is retained and resolved with any overlapping lower layer features, otherwise the overlapping lower layer representation is carried through unmodified. For a more detailed description, see implementation.

*DMRscaler* provides a solution to the problem of identifying DMR features across the full range of epigenetic feature sizes, whether at the basepair level or across entire chromosomes. The integration of results across iterations of the windowing procedure *DMRscaler* implements is a novel mechanism for defining DMRs that could be generalized to other epigenetic features or one dimensional data where discontinuity in components defining a feature of interest is expected and where features of interest may exist at dramatically different scales.

### Comparison of DMRscaler with existing methods

We next benchmarked *DMRscaler* to three commonly used methods in identification of differentially methylated regions: *bumphunter* [44], *comb-p* [45], and *DMRcate* [46] (Table 1). One significant difference between these methods is that our method, *DMRscaler,* and *comb-p* take pre-computed p-values as input while *bumphunter* and *DMRcate* use a t-test to determine individual level CpG significance. We observed that when running with a small sample size (n = 8 per group) there is poor correlation between the significance of differential methylation determined by the non-parametric Wilcoxon and t-test (Additional file 1: Figure S7). Since one of our goals was to develop a method that could detect DMRs in studies that compare rare disease datasets, the flexibility to choose the most appropriate statistical test for individual CpG significance based on experimental design and sample size constraints was desirable. While the t-test is appropriate when the sample size is sufficiently large (n > 30) or the sampling distribution is approximately normal, differential methylation analysis in small samples breaks these assumptions and therefore in our analysis of rare disease datasets the flexibility to use the Wilcoxon test was important.

**Table 1:** Comparison of differential methylation methods

	DMRscaler	bumphunter	comb-p	DMRcate
Individual CpG significance	NA (takes p-value as input)	t-test	NA (takes p-value as input)	- t-test, using M transformed Beta values
DMRs definition	Iterative enrichment testing for significant CpGs within window	Consecutive CpGs above significance threshold	Groups significant CGs if within window or window interval	Gaussian smoothed regions above significance threshold
DMR significance	Hypergeometric Test	Stouffer’s Method	Stouffer-Liptak	Permutation Test
Parameters controlling size (default)	window_size (4,8,16,32,64 adj CpGs)	maxGap (500 bp)	dist, step (no default)	lambda (1 kb), C (2)

The second difference between the differential methylation callers compared here (Table 1) lie in their modeling to identify differentially methylated regions. Briefly, *bumphunter* uses a linear regression model to identify CpG sites that are differentially methylated between case and control conditions. Then to detect DMRs, *bumphunter* identifies stretches of adjacent CpGs that are above a specified significance threshold after smoothing. However, the methylation landscape of adjacent CpGs is complex, with CpGs with high, intermediate and low β values mixed together making definition of large and contiguous regions of differential methylation challenging (Additional file 1: Figs. S4, S5). *Comb-p* uses the Stouffer-Liptak method for p-value correction and then groups significant CpGs within a window or window interval defined by the *dist* and *step* parameters. *DMRcate* is similar to *bumphunter* in that it also implements linear modeling (Table 1). *DMRcate* uses a Guassian smoothing function on M transformed β values to identify DMRs in genome-wide data. This provides the user with control of a bandwidth parameter, lambda, and control parameter, C, that can be used to identify larger regions of differential methylation. However, the behavior of *DMRcate* at larger bandwidth is poorly defined and the size of DMRs returned tends to be sensitive to parameter choice. For an in-depth review of methods see [48].

The design of the *DMRscaler* method has several unique features that allow it to more accurately identify larger co-regulated regions. First, it deals with the intrinsic variability in methylation distribution across the genome by binning adjacent CpGs into windows before assigning significance. Second, *DMRscaler* integrates the results from layers of windows defined with a series of window sizes to consider regions that are dramatically different in scale as potential regions of differential methylation. To accommodate a variety of study designs and constraints, *DMRscaler* operates on pre-computed *p*-values for individual level CpG significance. While here we use the Wilcox test due to the small sample size of our rare disease cohorts, other methods of generating *p*-values can be used, for instance to model the effect of covariates. Together, these features allow for the robust detection of differentially methylated regions across a large dynamic range, spanning basepair to megabase resolution and allow for detection of novel regions that are differentially methylated in rare disease cohorts and between other biological conditions such as chromosomal sex.

### *DMRscaler* accurately captures the scale of epigenetic features from basepair (bp) to megabase (Mb) size in simulated methylation data

Except where stated otherwise, in the following sections DMRs are assumed to be those in the most inclusive top layer, Layer 5, which is built using all lower layers and is meant to be the most accurate representation of DMR features.

To benchmark our method against existing methods, we compared *DMRscaler* to *bumphunter, comb-p,* and *DMRcate* on several metrics that highlight behavior of calling DMRs across a wide range of simulated DMR sizes. These metrics include: the correlation between simulated DMRs and DMRs called by each method across a wide range of simulated DMR sizes, the mapping value or the degree to which each method was able to represent individual simulated DMRs as single unified features, and the run time of each method. Additionally we used standard metrics of evaluation such as precision, recall, specificity, F1, Matthew’s correlation coefficient (MCC), and area under the precision-recall curve (AUCPR) to assess method performance.

We first simulated DMRs in methylation data from control blood samples (GSE74432) [10] ranging in size from 100 bp to 1 Mb (Fig. 2A, see method for details). In our simulation, we modeled the situation observed in real data where neighboring CpGs often have distinct methylation states with a *noise* parameter that represents the proportion of CpGs within simulated DMRs, excluding the first and last CpGs, that are left non-differentially methylated between the randomly selected samples placed in Group1 and Group2. In our simulations we tested *noise* parameter values of 0%, 25% and 50%. The *Δβ* parameter was used to control the magnitude of differential methylation with simulated DMRs, where *Δβ* is the difference in methylation proportion at non-noise CpGs introduced between the samples in Group1 and Group2. Simulations were run with the *Δβ* parameter set to values of 0.1, 0.2, and 0.4 to model small, modest, and large effect sizes for differential methylation respectively. Results from simulations run with each combination of these parameters are included in Additional file 1: Fig. S8, S9 and S10 and in Additional file 7: Table S9. The relative performance and behavior of methods was consistent across simulations run with each of these parameter combinations, so for space in the main text and figures we display results and report metrics from simulations run using *noise* = 50% and *Δβ* = 0.2.

*DMRscaler* was able to accurately call the size of the simulated DMRs (pearson’s r = 0.94) relative to *bumphunter* (pearson’s r = 0.04), *comb-p* (pearson’s r = 0.69), and *DMRcate* (pearson’s r = 0.85) (Fig. 2B). *DMRscaler* preserves a strong 1-to-1 relation between simulated and called DMRs, with 85% of simulated DMRs accurately called by *DMRscaler* with a 1-to-1 relation, compared with 19% for *bumphunter*, 44% for *comb-p*, and 69% for *DMRcate* (Fig. 2C).

To measure performance of our differential methylation caller, we calculate the AUCPR for each test. AUCPR combines a measure of precision of features called (ratio of true feature called to all features called) and recall (ratio of true features called to total number of true features) into a single value, with AUCPR = 0 representing no classification and AUCPR = 1 representing perfect classification of all features with no false positives. In our simulation, *DMRscaler* had an AUCPR of 0.79, *bumphunter* had an AUCPR value of 0.11, *comb-p* had an AUCPR of 0.34, and *DMRcate* had an AUCPR of 0.65 (Fig. 2D, see methods for details on AUCPR calculation). The low AUCPR of *bumphunter* is consistent with the low, slightly negative correlation observed between simulated and called DMR regions (Fig. 2B). This weak correlation is due to the fact that *bumphunter* has a strict requirement that significant differentially methylated CpGs are adjacent in order to belong to a common DMR and therefore breaks up simulated DMR features into many smaller features. The low AUCPR of *comb-p* is the result of a low recall rate of features that are much smaller than the size set by the *dist* and *step* parameters. Setting lower values for the *dist* parameters increases the ability to detect smaller DMR features but at the expense of detecting larger DMR features (Additional file 1: Figure S4), and at very large values for *dist* the run time becomes prohibitive especially as smaller step values are used (Additional file 1: Figure S4). *DMRcate* had a reasonably high AUCPR, however there is a bias in the size of DMRs called based on the choice of the bandwidth parameter lambda, and the control parameter C. Specifically, there is an excess of false calls of DMRs around 1 Mb and 1 kb (Fig. 2B and Additional file 1: Fig. S11) which is related to the choice of bandwidth parameter λ (set to 1 Mb) and the scaling parameter C (ratio of λ/C set to 500) (Additional file 1: Fig. S12). Our data suggests that *DMRcate* is able to identify larger DMRs but also that called DMR size is sensitive to parameter choice for the lambda and C parameters. This is supported by the shape of the precision-recall curve for *DMRcate* that shows a modest drop in precision as recall increases, suggesting *DMRcate* incurs a steeper false positive penalty compared with *DMRscaler*.

While the Wilcoxon test was used to generate p-values for individual level CpG significance for the simulation and real data analysis, we note that the performance and behavior of *DMRscaler* in the simulation was comparable when the T-test was used (Additional file 1: Fig. S13). Additionally, while these results focus on the top layer of results from *DMRscaler*, behavior at each lower layer is shown in Additional file 1: Fig. S14.

Comparing each method on each combination of Δβ (0.1, 0.2, and 0.4) and noise (0%, 25%, and 50%) parameters on metrics of precision, recall, specificity, F1, MCC, and AUCPR, *DMRscaler* consistently outperformed competing methods on each metric, except specificity where bumphunter was consistently the best performing method though the difference between methods on specificity was generally small (Table 2, Additional file 7: Table S9). These results further demonstrate that *DMRscaler* performs well for accurately calling DMRs across a wide range of feature size.

**Table 2:** Feature level evaluation of methods in simulation on proportion of CpGs differentially methylated in DMRs, precision, recall, specificity, F1, RCC, and AUCPR metrics on several choices of noise and Δβ parameters

Method	Δβ	noise	Proportion CGs Diff Methylated	Precision = 1-FDR (± 1 SD)	Recall (± 1 SD)	Specificity (± 1 SD)	F1 (± 1 SD)	MCC (± 1 SD)	AUCPR (± 1 SD)
bumphunter	0.1	0	0.56(± 0.1)	0.56(± 0.1)	0.06(± 0.03)	**1(± 6e-04)**	0.11(± 0.04)	0.14(± 0.04)	0.033(± 0.02)
comb-p	0.1	0	0.77(± 0.1)	0.71(± 0.1)	0.31(± 0.1)	0.98(± 0.01)	0.41(± 0.1)	0.45(± 0.09)	0.255(± 0.1)
DMRcate	0.1	0	0.78(± 0.2)	0.77(± 0.2)	0.67(± 0.1)	0.95(± 0.05)	0.69(± 0.07)	0.68(± 0.06)	0.58(± 0.07)
DMRscaler	0.1	0	**0.99(± 0.02)**	**0.98(± 0.02)**	**0.89(± 0.03)**	**1(± 0.006)**	**0.94(± 0.02)**	**0.93(± 0.02)**	**0.885(± 0.03)**
bumphunter	0.2	0	0.76(± 0.2)	0.76(± 0.2)	0.5(± 0.03)	**1(± 8e-04)**	0.59(± 0.05)	0.36(± 0.04)	0.409(± 0.08)
comb-p	0.2	0	0.64(± 0.3)	0.61(± 0.3)	0.2(± 0.1)	0.97(± 0.004)	0.3(± 0.2)	0.33(± 0.2)	0.161(± 0.1)
DMRcate	0.2	0	0.77(± 0.02)	0.77(± 0.03)	0.82(± 0.03)	0.92(± 0.01)	0.8(± 0.009)	0.76(± 0.01)	0.695(± 0.03)
DMRscaler	0.2	0	**0.99(± 0.008**)	**0.99(± 0.009)**	**0.94(± 0.02)**	0.99(± 0.006)	**0.96(± 0.01)**	**0.96(± 0.01)**	**0.932(± 0.02)**
bumphunter	0.4	0	0.89(± 0.1)	0.89(± 0.1)	0.51(± 0.02)	**1(± 4e-04)**	0.65(± 0.04)	0.35(± 0.02)	0.507(± 0.03)
comb-p	0.4	0	0.78(± 0.05)	0.71(± 0.07)	0.29(± 0.1)	0.98(± 0.008)	0.4(± 0.08)	0.44(± 0.06)	0.239(± 0.08)
DMRcate	0.4	0	0.81(± 0.03)	0.82(± 0.04)	0.81(± 0.08)	0.94(± 0.03)	0.81(± 0.03)	0.78(± 0.02)	0.704(± 0.06)
DMRscaler	0.4	0	**0.99(± 0.008)**	**0.99(± 0.008)**	**0.97(± 0.02)**	0.99(± 0.007)	**0.98(± 0.009)**	**0.97(± 0.01)**	**0.956(± 0.02)**
bumphunter	0.1	0.25	0.35(± 0.2)	0.35(± 0.2)	0.012(± 0.01)	**1(± 6e-04)**	0.023(± 0.02)	0.053(± 0.03)	0.003(± 0.003)
comb-p	0.1	0.25	0.68(± 0.02)	0.83(± 0.02)	0.36(± 0.1)	0.98(± 0.006)	0.5(± 0.1)	0.53(± 0.08)	0.337(± 0.1)
DMRcate	0.1	0.25	0.71(± 0.03)	0.88(± 0.03)	0.54(± 0.06)	0.96(± 0.03)	0.67(± 0.04)	0.67(± 0.02)	0.508(± 0.04)
DMRscaler	0.1	0.25	**0.83(± 0.007)**	**0.99(± 0.008)**	**0.86(± 0.05)**	0.99(± 0.006)	**0.92(± 0.03)**	**0.92(± 0.03)**	**0.848(± 0.06)**
bumphunter	0.2	0.25	**0.87(± 0.1)**	0.87(± 0.1)	0.32(± 0.03)	**1(± 9e-04)**	0.47(± 0.03)	0.24(± 0.03)	0.268(± 0.05)
comb-p	0.2	0.25	0.49(± 0.3)	0.59(± 0.3)	0.26(± 0.1)	0.97(± 0.01)	0.35(± 0.2)	0.37(± 0.2)	0.226(± 0.1)
DMRcate	0.2	0.25	0.66(± 0.03)	0.82(± 0.04)	0.76(± 0.07)	0.94(± 0.03)	0.79(± 0.03)	0.76(± 0.02)	0.672(± 0.05)
DMRscaler	0.2	0.25	0.82(± 0.02)	**0.97(± 0.02)**	**0.85(± 0.03)**	**1(± 0.004)**	**0.91(± 0.02)**	**0.91(± 0.02)**	**0.841(± 0.02)**
bumphunter	0.4	0.25	**0.96(± 0.05**)	0.96(± 0.05)	0.32(± 0.01)	**1(± 7e-04)**	0.48(± 0.01)	0.19(± 0.02)	0.32(± 0.01)
comb-p	0.4	0.25	0.65(± 0.04)	0.79(± 0.06)	0.25(± 0.09)	0.98(± 0.01)	0.37(± 0.09)	0.43(± 0.05)	0.215(± 0.08)
DMRcate	0.4	0.25	0.66(± 0.03)	0.82(± 0.03)	0.8(± 0.09)	0.93(± 0.04)	0.8(± 0.04)	0.78(± 0.02)	0.682(± 0.06)
DMRscaler	0.4	0.25	0.83(± 0.008)	**0.99(± 0.006)**	**0.85(± 0.03)**	0.99(± 0.006)	**0.92(± 0.02)**	**0.92(± 0.02)**	**0.844(± 0.03)**
bumphunter	0.1	0.5	0.15(± 0.1)	0.16(± 0.1)	0.004(± 0.001)	**1(± 8e-04)**	0.007(± 0.002)	0.017(± 0.007)	0.001(± 3e-04)
comb-p	0.1	0.5	0.5(± 0.07)	0.83(± 0.1)	0.32(± 0.04)	0.97(± 0.01)	0.46(± 0.05)	0.5(± 0.05)	0.288(± 0.07)
DMRcate	0.1	0.5	0.55(± 0.02)	0.91(± 0.02)	0.41(± 0.05)	0.99(± 0.01)	0.56(± 0.05)	0.6(± 0.03)	0.39(± 0.05)
DMRscaler	0.1	0.5	**0.63(± 0.02)**	**1(± 0.004)**	**0.76(± 0.01)**	0.99(± 0.004)	**0.86(± 0.008)**	**0.86(± 0.008)**	**0.754(± 0.01)**
bumphunter	0.2	0.5	**0.93(± 0.05)**	0.93(± 0.05)	0.18(± 0.01)	**1(± 4e-04)**	0.31(± 0.02)	0.17(± 0.01)	0.169(± 0.01)
comb-p	0.2	0.5	0.52(± 0.02)	0.86(± 0.02)	0.35(± 0.04)	0.98(± 0.007)	0.49(± 0.03)	0.54(± 0.02)	0.33(± 0.03)
DMRcate	0.2	0.5	0.52(± 0.02)	0.9(± 0.04)	0.59(± 0.1)	0.98(± 0.03)	0.7(± 0.06)	0.71(± 0.04)	0.565(± 0.09)
DMRscaler	0.2	0.5	0.64(± 0.02)	**0.99(± 0.008)**	**0.81(± 0.02)**	0.99(± 0.006)	**0.89(± 0.01)**	**0.89(± 0.01)**	**0.797(± 0.02)**
bumphunter	0.4	0.5	**0.9(± 0.08)**	0.9(± 0.08)	0.17(± 0.02)	**1(± 7e-04)**	0.29(± 0.02)	0.16(± 0.02)	0.173(± 0.01)
comb-p	0.4	0.5	0.51(± 0.04)	0.85(± 0.03)	0.37(± 0.04)	0.99(± 0.01)	0.51(± 0.04)	0.55(± 0.03)	0.351(± 0.04)
DMRcate	0.4	0.5	0.5(± 0.02)	0.85(± 0.03)	0.74(± 0.09)	0.96(± 0.02)	0.79(± 0.04)	0.77(± 0.03)	0.679(± 0.06)
DMRscaler	0.4	0.5	0.63(± 0.01)	**0.99(± 0.009)**	**0.79(± 0.009)**	0.99(± 0.007)	**0.88(± 0.008)**	**0.88(± 0.009)**	**0.78(± 0.01)**

Noise is the proportion of CpGs within a simulated DMR that are not differentially methylated, Δβ is the difference in the proportion of methylation introduced at non-noise CpGs within the simulated DMR region. Shown are the mean values across five replicates for each measure and the standard deviation. Bolded are the best performing method for the given combination of noise and Δβ parameters

While *DMRscaler* performs well compared to other methods at the task of identifying DMRs across a wide range of scales, the method also performs well in terms of computational time to the other methods that are time efficient with larger window size analogous parameters. On average *DMRscaler* required 30 s to 1 min to complete a run, *bumphunter* required around 1 to 3 min, and *DMRcate* only required around 10 s to call DMRs. *Comb-p,* which uses a sliding window mechanism similar to *DMRscaler,* required an hour to complete each run with the given parameter set (Fig. 2E).

The simulation results show that *DMRscaler* reconstructs the scale of DMR features more accurately than other methods across a wide range of DMR feature sizes as measured by called and simulated DMR size correlation, mapping value, and precision and recall. Additionally on other measures of performance including specificity, F1, MCC, and AUCPR, *DMRscaler* consistently performs well compared to other methods on simulated datasets with DMRs that vary widely in terms of scale.

### Differential methylation between 46, XX and 46, XY individuals captures chromosome-wide and gene specific regulatory features in empiric data.

**Fig. 3:**
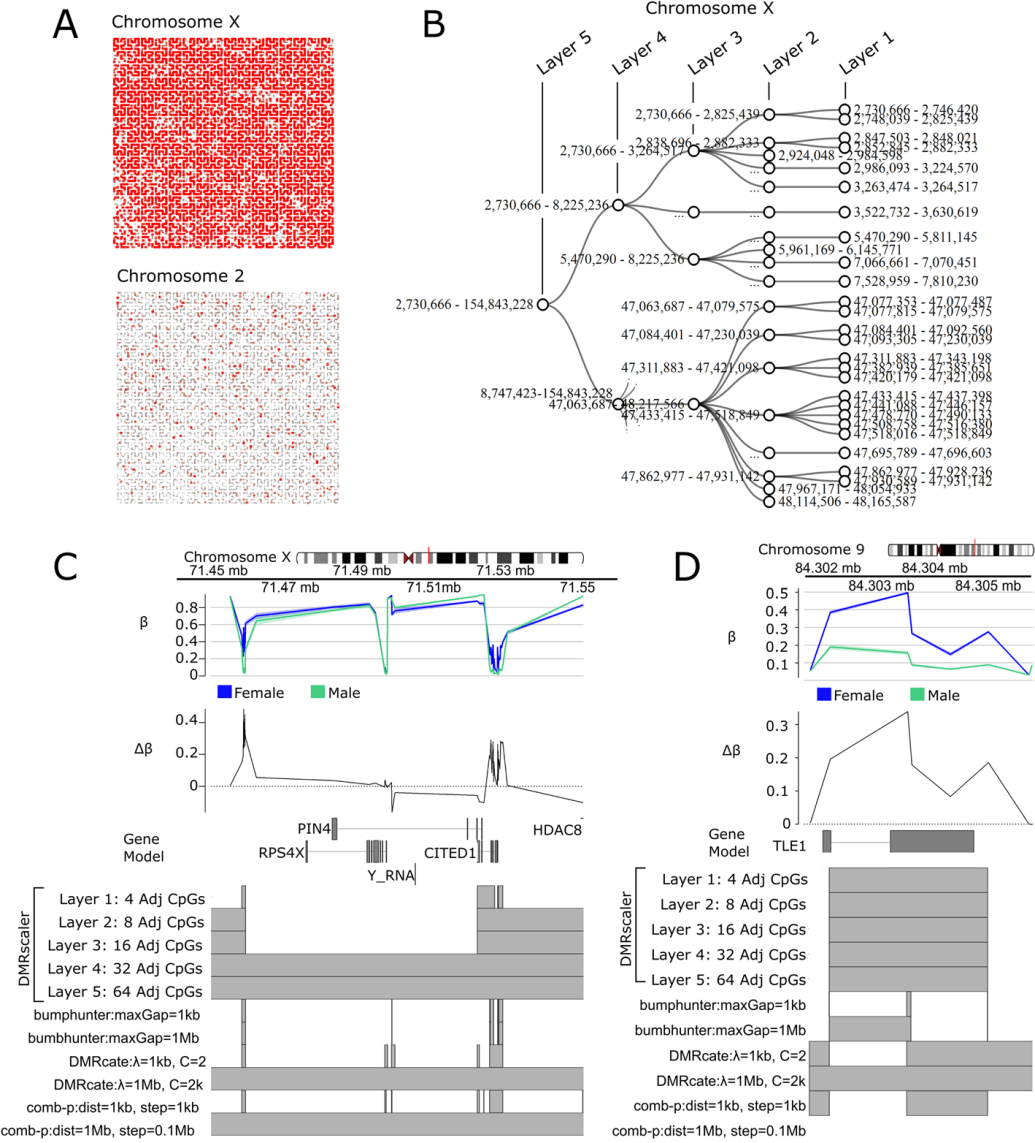
Differential Methylation Analysis Between XX and XY individuals. **A** Hilbert Curves of chrX and chr1. Hilbert curve is constructed by ordering CpGs by their position along the given chromosome. Red points are differentially methylated CpGs with FDR < 0.1. Point size scaled to max significance level (-log10 p-value). **B** Diagonal network plots showing hierarchical relation of DMRs called by *DMRscaler* in Layers 4, 3, 2, and 1 (equivalent to 32 16, 8, and 4 Adj. CpG Layers respectively) for X-chromosome. **C** ChrX:71.4–71.6 Mb. GVIZ track stack plot. Top track shows mean β value per group, next track shows Δβ, where Δβ = β_female_−β_male_. Below the gene model track is the DMR track, highlighting the regions called as a DMR at each result layer from *DMRscaler* (Layers 1, 2, 3, 4, 5 are equivalent to 4, 8, 16, 32, 64 Adj. CpG layers) and from each competing method. (D) Chr9:84.302–84.306 Mb. Tracks same as 3C

To test our hypothesis in real-world DNA methylation data, we sought to determine whether our method could capture both small regions of autosomal differential methylation as well as chromosome-wide features such as X-chromosome inactivation. Therefore, our test case is the natural occurrence of X-inactivation in females, where one copy of the X-chromosome is largely inactivated by the action of the lncRNA *Xist* [18, 19]. This process of inactivation is correlated with a striking chromosome-wide difference in DNA methylation between males and females on the X-chromosome (Fig. 3A, top) as compared to the autosomes, e.g. chromosome 2 where the size of differentially methylated regions span 103 bp to 873 bp (Fig. 3A, bottom, Additional file 2: Table S2). Across all chromosomes, the size of DMRs called by *DMRscaler* spans 98 bp to 152 Mb, representing a 1.5 million-fold difference in the scale of DMRs detected by *DMRscaler*.

With the visual intuition of the scale of differential methylation between sexes from Fig. 3A, we next compared the result of differential methylation analysis using *DMRscaler*, *bumphunter*, *comb-p* and *DMRcate*. *DMRscaler* was the only method that consolidated the observed differential methylation into a single DMR that spanned 98% of the X-chromosome (Table 3, Fig. 3B, Additional file 1: Fig. S15). Even with the maxWidth parameter set to 1 Mb, *Bumphunter* reported 1,162 unique DMRs on the X-chromosome with a median width of 531 bp (IQR: 1 bp–1.21 kb) (Additional file 1: Fig. S16, Additional file 2: Table S2), likely due to a lack of mechanism for spanning non-differentially methylated CpGs. With a standard parameter set of dist = 1 kb, step = 100 bp, *comb-p* reported 2,390 unique DMRs on the X-chromosome with a median width of 2 bp (IQR: 2 bp–963 bp) (Table 3, Additional file 1: Fig. S16, Additional file 2: Table S2). With a wider parameter set of dist = 1 Mb, step = 100 kb, *comb-p* called 19 unique DMRs on the X-chromosome with a median width of 3.15 Mb (IQR: 512 kb–8.54 Mb) (Table 3, Additional file 1: Fig. S16, Additional file 2: Table S2). *DMRcate* with default settings reported 1178 unique DMRs on the X-chromosome with a median width of 1.09 kb (IQR: 616 bp–1.68 kb). When *DMRcate* was provided with a larger bandwidth parameter (lambda = 1 Mb, C = 2000) it improved in consolidating the DMRs, but still reported 15 unique DMRs (median width: 3.95 Mb, IQR: 1.00 Mb–17.89 Mb). For complete distributions of called DMR sizes see Additional file 1: Fig. S17, Additional file 2: Table S2.

**Table 3:** Sex analysis DMR summary table

Method	Chromosome X				Autosomes			
	# DMRs	Mean DMR width	Median DMR width	% total width	# DMRs	Mean DMR width	Median DMR width	% total width
*DMRscaler*								
Layer 1: 4 adj CpGs	694	67.91 kb	6.29 kb	30	18	616 bp	398 bp	0.00038%
Layer 2: 8 adj CpGs	246	381.32 kb	114.54 kb	61	19	935 bp	494 bp	0.00062%
Layer 3: 16 adj CpGs	20	7.30 Mb	1.54 Mb	94	21	4.73 kb	587 bp	0.0035%
Layer 4: 32 adj CpGs	2	75.80 Mb	75.80 Mb	98	23	19.59 kb	624 bp	0.016%
Layer 5: 64 adj CpGs	1	152.11 Mb	152.11 Mb	98	22	20.32 kb	606 bp	0.016%
*bumphunter*								
Default: maxGap = 1 kb kb	1258	527 bp	238 bp	0.43	32	67 bp	1 bp	0.000075%
maxGap = 1 Mb	1162	6.76 kb	531 bp	5.1	32	611 bp	1 bp	0.00068%
*comb-p*								
dist = 1 kb, step = 100 bp	2390	567 bp	2 bp	0.87	580	330 bp	292 bp	0.0067%
dist = 1 Mb,step = 100 kb	19	6.13 Mb	3.15 Mb	75	29	140.59 kb	127.23 kb	0.14%
*DMRcate*								
Default:lambda = 1 kb, C = 2	1178	1.30 kb	1.09 kb	0.99	826	658 bp	558 bp	0.019%
Lambda = 1 Mb, C = 2000	15	8.53 Mb	3.95 Mb	83.0	197	7.63 kb	676 bp	0.052%

*DMRscaler* iteratively calls DMR-like regions using windows of increasing size while integrating the results of each iteration into the next layer of DMRs. While the top-most layer is the primary output of *DMRscaler*, this procedure produces a nested hierarchy of DMRs when considering the list of results across all layers that allows for a nuanced view of the differential methylation architecture. In Fig. 3B, a subset of this hierarchy within the X-chromosome is shown. Here DMRs called at layer 1 are consolidated in the DMRs in layer 2, and then those DMRs in layer 2 are consolidated into DMRs in layer 3, consolidation of DMRs in layer 3 into layer 4 results in the final consolidation of DMRs into a single feature spanning the entire X-chromosome (Fig. 3B).

While the entirety of the X-chromosome can be considered a differentially methylated feature, it has been well established that there is a small subset of genes on the X-chromosome that escape X-inactivation and DNA methylation [49]. The expectation when comparing methylation between females and males is that X-inactivation would result in differential methylation between sexes, with hypermethylation and to a lesser extent hypomethylation across the entire X-chromosome in females compared to males [50]. Therefore, we expect regions where the Δβ between the two groups is at or near zero would be enriched in regions that escape X-inactivation due to a relative lack of differential methylation at these sites. An example of one such region is the gap of two DMRs that persists until the integration between layer 3 and layer 4 which occurs at chrX: 71,459,274–71,521,494 which corresponds to the gene *RPS4X* (Fig. 3C, S18), which is known to escape X-inactivation [51]. To test whether this trend of gaps in DMRs mapping to held more generally across regions escaping X-inactivation, we performed an enrichment test for CpGs that overlapped genes known to escape X-inactivation and CpGs overlapping gaps in DMRs called at each layer of *DMRscaler*'s output. A consensus of genes known to escape or be silenced in X-inactivation reported in a 2015 study by Balaton et al. was used for the enrichment test [52]. In layers 1, 2, 3, and 4 which were defined by windows of 4, 8, 16, and 32 adjacent CpGs respectively, the odds ratio between CpGs overlapping gaps between DMRs and CpGs overlapping genes that escape X-inactivation were OR = 7.57 (95% CI 6.38–8.99; *p*-value = 1.04e-134 Fisher’s exact test), OR = 7.24 (95% CI 6.07–8.65; *p*-value = 5.93e-100 Fisher’s exact test), OR = 51.99 (95% CI 30.38–90.33; *p*-value = 4.34e-77 Fisher’s exact test), and OR = 160.44 (95% CI 25.42–6,396.92; *p*-value = 5.93e-100 Fisher’s exact test) respectively. At layer 5 no enrichment was detected, as the whole X-chromosome was consolidated into a single feature. *Bumphunter* similarly displayed substantial enrichment, with odds ratios estimated between OR ≈ 10–20, however, as noted earlier, bumphunter could not consolidate DMRs on the X-chromosome to identify the whole of the X-chromosome as differentially methylated. *Comb-p* and *DMRcate* each observed much smaller associations of gaps between DMRs and genes escaping X-inactivation with ORs ≈ 1–3 (Table 4). These results demonstrate that while the top layer DMR, which spans the X-chromosome, correlates most intuitively with the phenomenon of X-inactivation, the exploration of the hierarchical structure of complex DMRs that is enabled by *DMRscaler* can reveal biologically meaningful features such as patterns of genic escape from X-inactivation.

**Table 4:** Enrichment test for association between genes silenced by X-inactivation and DMRs, and genes that escape from X-inactivation and gaps between DMRs. Only CpGs on the X-chromosome overlapping genes are used in the enrichment test

Method	Odds ratio (95% CI)	*p*-value (Fisher’s Exact)
*DMRscaler*		
Layer 1: 4 adj CpGs	7.57 (6.38–8.99)	1.04e-134
Layer 2: 8 adj CpGs	7.24 (6.07–8.65)	5.93e-100
Layer 3: 16 adj CpGs	51.99 (30.38–90.33)	4.34e-77
Layer 4: 32 adj CpGs	160.44 (25.42–6,396.92)	6.59e-18
Layer 5: 64 adj CpGs	0 (0–319.59)	1
*bumphunter*		
Default: maxGap = 1 kb	14 (11.18–17.61)	7.00E-185
maxGap = 1 Mb	15 (11.95–18.94)	5.36E-193
*comb-p*		
dist = 1 kb, step = 100 bp	2.07 (1.63–2.62)	5.72E-09
dist = 1 Mb, step = 100 kb	0 (0–Inf)	1
*DMRcate*		
Default: lambda = 1 kb, C = 2	1.27 (1.03–1.57)	0.023608
Lambda = 1 Mb, C = 2000	0 (0–43.86)	1

The complex hierarchical relation of DMRs within the X-chromosome contrasts with the DMRs of the autosomal chromosomes. DMRs on the autosome show little to no branching, which implies that these DMRs are stable at each iteration of the algorithm (Additional file 1: Fig. S15). A genome view of one such DMR at chr9: 84,302,344–84,304,414 highlights this stability, where a feature identified as a DMR at the first layer of the algorithm is stable through each subsequent iteration (Fig. 3D, Additional file 1: Fig. S18). The gene *TLE1* overlaps this DMR and has previously been identified as an autosomal gene that is differentially methylated between males and females [53, 54].

The results of the differential methylation analysis between sexes highlights the utility of *DMRscaler* in identifying differential methylation features that exist at dramatically different scales in real data. This ability distinguishes *DMRscaler* from existing methods which either are unable to identify larger DMRs while preserving the stability of smaller DMRs, as in *DMRcate* and *comb-p*, or tend to fragment larger DMR into many smaller features, as in *bumphunter*. A brief analysis of the hierarchical structure that results from *DMRscaler*'s layer merging mechanism reveals how *DMRscaler* can capture biologically meaningful structure within a DMR, such as escape from X-inactivation. This ability to represent DMR structure more completely highlights *DMRscaler*’s potential value as a tool for exploring the interactions between features of epigenetic regulation at different scales.

### Rare chromatin modifier syndromes contain regions of differential methylation spanning gene clusters critical for development

Next we analyzed DNA methylation datasets from several rare diseases of chromatin modifier genes to see whether *DMRscaler* revealed novel DMR features that might otherwise be missed by existing methods. Except where stated otherwise, in the following sections DMRs are assumed to be those in the most inclusive top layer, Layer 5, which is built using all lower layers and is meant to be the most accurate representation of DMR features.

**Fig. 4:**
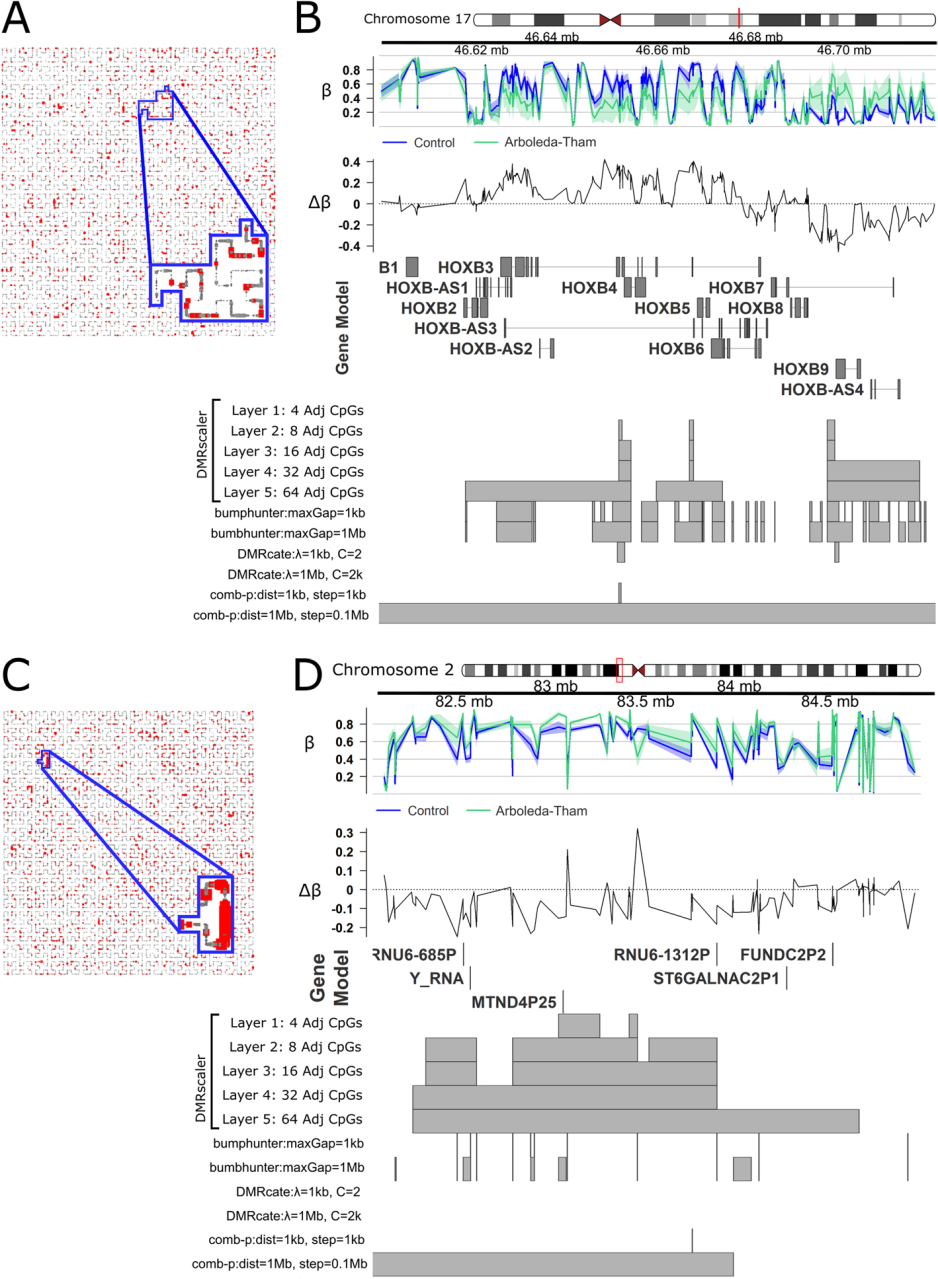
Differential Methylation Analysis in Arboleda-Tham Syndrome. **A** Hilbert curve of CpGs from chr17, outlined is the region corresponding to Chr17:46.59–46.73 Mb, the *HOXB* cluster. CpGs with FDR < 0.1 are highlighted red. Point size is scaled to maximum significance value. **B** Chr17:46.59–46.73 Mb. *HOXB* cluster. GVIZ track stack plot. Top track shows mean β value per group, next track shows Δβ, where Δβ = β_Control_−β_Arboleda-Tham_. Below the gene model track is the DMR track, highlighting the regions called as a DMR at each result layer from *DMRscaler* and from each competing method. **C** Chr2:81.5–84.5 Mb. Design same as 4A. **D** Chr2:81.5–84.5 Mb. Tracks same as 4B

First, we compared the DNA methylation profile from fibroblasts from Arboleda-Tham syndrome patients to control samples. This analysis consisted of 20 samples, with 8 patients and 12 controls (Additional file 1: Table S1). All patients were previously reported by Kennedy, et al. [7]. In our analysis, *DMRscaler* identified 390 unique DMRs with a median width of 144.59 kb (IQR: 21.1 kb–481.2 kb), resulting in a total genomic coverage of 4.9% (151.35 Mb) (Additional file 1: Table S10, Fig. S19). Over the *HOXB* gene cluster three unique DMRs were identified. The first and second DMRs overlap regions of *HOXB2, HOXB3* and *HOXB4* and is hypomethylated in Arboleda-Tham Syndrome patients relative to controls. The second overlaps part of *HOXB5 and HOXB6* and is also hypomethylated in Arboleda-Tham Syndrome patients. The third spans *HOXB9* and is hypermethylated in Arboleda-Tham Syndrome patients relative to controls (Fig. 4A,B, Additional file 1: Fig. S20). *Bumphunter* calls many more DMRs over this region that are highly fragmented, including regions missed by *DMRscaler*. This is likely due to the regions called by bumphunter having substantial variance that the Wilcox test used to pre-compute p-values for *comb-p* and *DMRscaler* being more conservative than the t-test used by bumphunter. *Comb-p* with the large distance parameter of 1 Mb calls the entire region as differentially methylated. The relatively large coverage of the genome by DMRs is driven primarily by multi-megabase scale DMRs identified spanning relatively gene sparse regions, which other methods are unable to consolidate, with the exception of *comb-p* using a distance parameter of 1 Mb (e.g. Figure 4C, D, Additional file 1: Fig. S20).

Weaver syndrome (MIM# 277590), is a rare overgrowth disorder that is caused by de novo mutations in *EZH2*, a histone methyltransferase. Comparing Weaver syndrome patient samples to controls, *DMRscaler* identified 226 unique DMRs with a median width of 8.88 kb (IQR: 1.92 kb–30.04 kb). These regions comprised a total of 0.40% (12.34 Mb) of the genome (Additional file 1: Table S11, Figure S21).

**Fig. 5:**
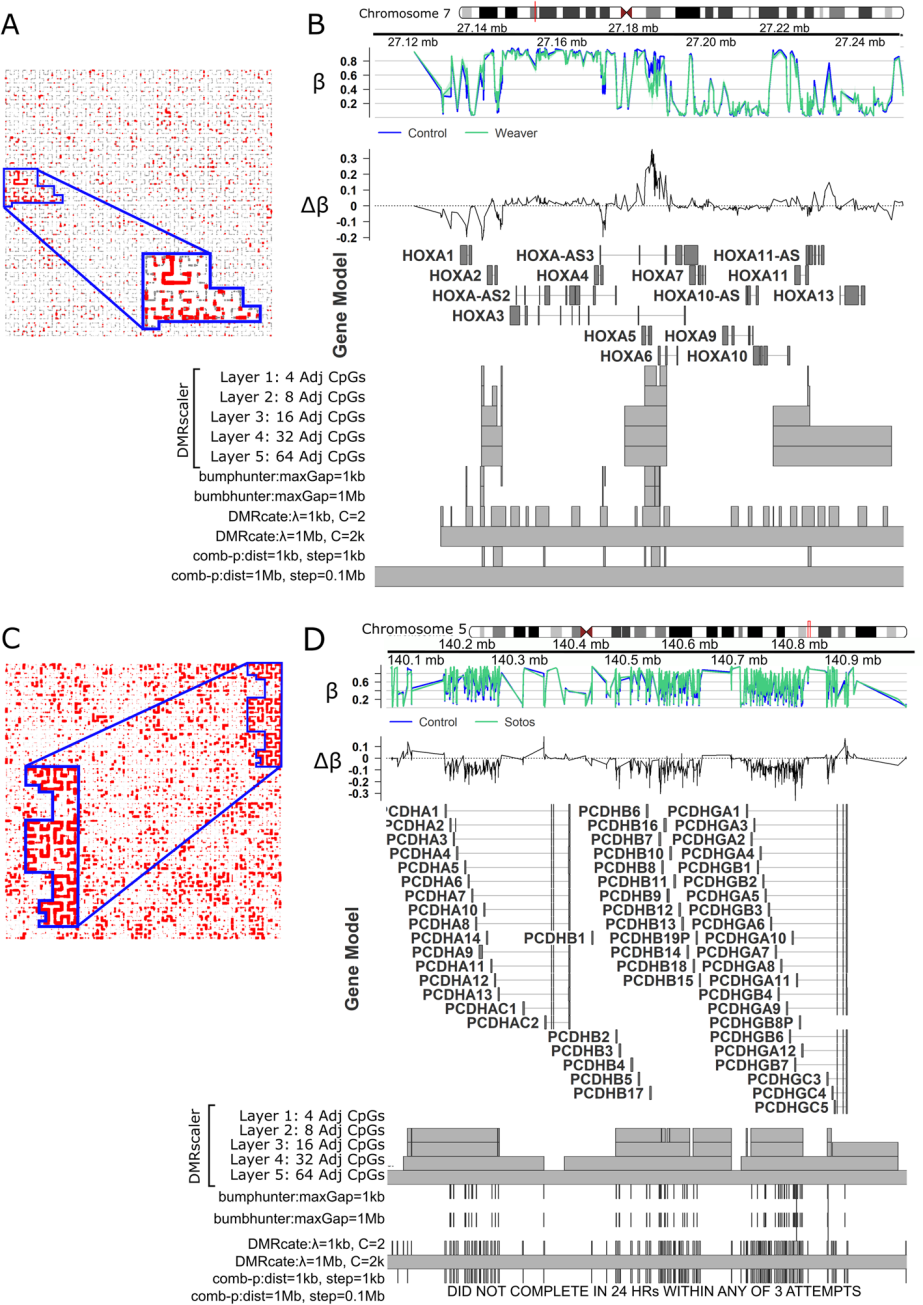
Differential Methylation Analysis in Weaver (A,B,C) and Sotos Syndrome (D,E,F). **A** Hilbert curve of CpGs from chr7, outlined is the region corresponding to Chr7:27.1–27.3 Mb, the *HOXA* cluster. CpGs with FDR < 0.1 are highlighted red. Point size is scaled to maximum significance value. **B** Chr7:27.1–27.3 Mb. *HOXA* cluster. GVIZ track stack plot. Top track shows mean β value per group, next track shows Δβ, where Δβ = β_Control_−β_Weaver_. Below the gene model track is the DMR track, highlighting the regions called as a DMR at each result layer from *DMRscaler* and from each competing method. **C** Chr5:140.1–140.8 Mb over the *PCDH* clusters. Design same as 4A. **D**.Chr5:140.1–140.8 Mb over the *PCDH* clusters. Tracks same as 4B. except that Δβ = β_Control_−β_Sotos_

Over the *HOXA* gene cluster, *DMRscaler* identified three distinct DMRs associated with Weaver Syndrome. The first spans *HOXA1-HOXA2* and is modestly hypermethylated in Weaver syndrome, the second covers *HOX5* and the last two exons of *HOX6* and is hypomethylated in Weaver syndrome cases relative to controls. The third DMR covers the first exon of *HOXA10,* as well as *HOXA11,* and *HOXA13*. This third DMR is generally weakly hypermethylated in Weaver Syndrome, with a small but significant region of hypomethylation just upstream of *HOXA11*. The other methods all report DMRs overlapping these clusters, however they are either fragmented or overly broad (Fig. 5A, B, Additional file 1: Fig. S22).

Finally, we also analyzed Sotos Syndrome (MIM# 117550), an overgrowth syndrome caused by truncating and missense mutation in the nuclear receptor binding SET domain protein 1 **(***NSD1)* gene [55]. Analysis with DMRscaler identified 1776 unique DMRs with a median width of 555.13 kb (IQR: 156 kb–1.40 Mb), covering 71% of the genome (2.17 Gb), a similar degree of coverage was seen with *DMRcate* where 282 DMRs spanned 77% of the genome (Additional file 1: Table S12, Fig. S23). We identified three unique DMRs at the 32 Adj CpG layer that span gene clusters of protocadherins. These DMRs caused by mutations in *NSD1* cover the neighboring *Protocadherin* (*PCDH*) gene cluster *PCDHA*, *PCDHB*, and *PCDHGB* (Fig. 5D, E, S22), which encode large transmembrane proteins that are critical for a diverse range of processes ranging from cell-signaling to dendritic arborization [56]. One DMR spans the first exons of *PCDHA1-PCDHA12*, another spans from *PCDHB2* to *PCDHB19P*, and the third covers the first exons of *PCDHGA3-PCDHGA12* and *PCDHB1-PCDHGC5*. All of the DMRs covering these *PCDH* clusters are hypermethylated in Sotos Syndrome relative to controls, though it is notable that the β values of CpGs across these clusters are highly variable reflecting an example of the neighboring CpG heterogeneity described earlier (Fig. 5D, Additional file 1: Figs. S22, S4, S5). Notably, only DMRcate with parameters λ = 1 Mb, and C = 2000 was also able to call a DMR over this region, however it lacks a mechanism to see the interior structure that shows that each of these three clusters is seperated by regions of non-differentially methylated CpGs that is captured by *DMRscaler*’s hierarchical output.

These results in rare chromatin modifier syndromes highlight *DMRscaler's* utility in identifying patterns of differential methylation that exist over broader genomic features such as gene clusters.

### Analysis of overlapping regions of differential methylation

Following analysis of each syndrome individually, we asked whether there was evidence of shared regions differentially methylated between Arboleda-Tham, Sotos, and Weaver syndrome. Between Arboleda-Tham and Sotos syndrome, we identified 652 regions with overlapping DMRs (77.3% of total DMRs for Arboleda-Tham, 4.7% of total DMRs for Sotos), and 458 genes overlapped by some DMR in both syndromes (11.4% of genes overlapping a DMR in Arboleda-Tham syndrome, 3.1% of genes overlapping a DMR in Sotos syndrome). Between Arboleda-Tham and Weaver syndrome, we identified 48 regions with overlapping DMRs (1.3% of total DMRs for Arboleda-Tham syndrome, 14.1% of total DMRs for Weaver syndrome), and 39 genes overlapped by some DMR in both syndromes (13.2% of genes overlapping a DMR in Arbolelda-Tham syndrome, 5.6% of genes overlapping a DMR in Weaver syndrome). Between the two growth disorders, Sotos and Weaver syndrome, we identified 414 regions (0.7% of total DMRs for Sotos, 91.0% of total DMRs for Weaver) and 282 genes overlapped by some DMR in both syndromes (5.9% of genes overlapping a DMR in Sotos, 93.1% of genes overlapping a DMR in Weaver) (Additional file 6: Table S6).

To test the significance of the overlap we tested the odds ratio (OR) of overlap between each pair of syndromes. To simplify the analysis and make the measure of the odds ratio closer in form to the *DMRscaler* method, we only used counts of measured CpGs (See methods for details). Essentially, the odds ratio tests whether there is enrichment of CpGs that are in DMRs in one syndrome in the set of CpGs found in DMRs in the other syndrome being compared. OR with a confidence interval (CI) overlapping 1 suggests no enrichment, closer to 0 or further from 1 indicates greater enrichment. The raw overlap counts used to calculate the OR are in (Additional file 1: Table S7) and the odds ratios are reported in (Additional file 1: Table S8). The highest odds ratio was between Sotos and Weaver with OR = 17.16 (95% CI 12.27–23.99, *p* = 1.9e-32 Fisher’s exact test) in Layer 1. The OR between Sotos and Weaver drops to OR = 1.55 (95% CI 1.47–1.64, *p* = 4.6e−56 Fisher’s exact test) in Layer 5, which is likely due to the extensive genomic coverage by DMR features in Sotos syndrome at Layer 5. The next highest odds ratio was between Arboleda-Tham and Weaver in Layer 1 with OR = 9.94 (95% CI 5.83–16.94, *p* = 4.7e-10 Fisher’s exact test) which gains in significance but drops in magnitude at Layer 5 with OR = 3.00 (95% CI 2.71–3.31, *p* = 3.1e-77 Fisher’s exact test). The OR between Arboleda-Tham and Sotos is relativately small at Layer 1 with OR = 1.72 (95% CI 1.43–2.06, *p* = 5.6e−8 Fisher's exact test) and drops to non-significance at Layer 5 (Additional file 1: Table S8). These results show how DMRs of each of the three syndromes analyzed here are enriched in CpGs in DMRs in each of the other syndromes tested here at the lower layers output by *DMRscaler*, where differential CpG density is required to be higher, and in particular the strong enrichment between the two overgrowth disorders offers evidence for common epigenetic effects in these disorder and potentially common contributing factors.

One region of overlap between Sotos and Weaver syndrome was a region overlapping *INS,* and *INS-IGF2* proximal to *IGF2*. This region stood out as a region implicated in another growth disorder, Beckwith-Wiedemann syndrome (BWS) [57]. The DMR called for Sotos syndrome is just upstream of *IGF2* and overlaps *INS* and *INS-IGF2*, with sites of moderate effect hypomethylation (Δβ > 0.2) (Additional file 1: Fig. S24A). The DMR called for Weaver syndrome overlaps the IGF2 gene and is composed of sites with a small effect size (Δβ ~ 0.05), with hypermethylation over a region of the *IGF2* gene body and hypomethylation further upstream overlapping the *INS* and *INS-IGF2* genes. Upstream of *IGF2* overlapping *INS* and *INS-IGF2* the pattern of hypomethylation in Sotos and Weaver syndrome was consistent (Additional file 1: Fig. S24B, Additional file 6: Table S6).

Across all three syndromes there were 49 genes overlapping some DMR called by *DMRscaler*. Among these were *PCDHGA1, PCDHGA2, PCDHGA3, PCDHGA8, PCDHGA10, PCDHGB7, PCDHGA11*, *PCDHGA12,* and *PCDHGC3* of the *PCDHG* cluster genes, previously discussed in context of Sotos syndrome alone. These genes are worth noting as they are involved in neural development. The *PCDHG* cluster is broadly hypermethylated in Sotos, as noted earlier (Fig. 5C, D). In Arboleda-Tham syndrome, there is a small DMR intergenic to most of the *PCDHG* genes in this cluster and positioned at the 5′ end of *PCDHGC3*. In Weaver syndrome there is a DMR with minor hypomethylation at the stretching from the same 5′ end of *PCDHGC3* Arboleda-Tham that spans through to the shared 3′ end of *PCDHG* genes. Few of the other methods evaluated are able to identify this region of the *PCDHG* gene cluster in either Arboleda-Tham syndrome or Weaver syndrome (Additional file 1: Fig. S25).

The overlapping DMRs and the genes with overlapping DMRs between Arboleda-Tham, Sotos, and Weaver syndrome reveal a number of shared regions of differential methylation and shared genes with patterns of differential methylation. CpGs in DMRs in any one syndrome are enriched in CpGs in DMRs of either of the other syndromes across all three pairs of syndromes, Arboleda-Tham:Sotos, Arboleda-Tham:Weaver, Sotos:Weaver, as measured by the odds ratio. However, these data are derived from different cell types (fibroblast vs blood) and exhibit cell-type specific changes in addition to those caused by the genetic mutation. Together these results suggest that while each syndrome has a distinct profile of differential methylation, there is also significant overlap in regions mirroring shared phenotypic features.

## Discussion

The key development of our new method, *DMRScaler*, is a substantial improvement over existing methods in the ability to accurately identify the size of DMRs across the full range of epigenetic scale.

Differential methylation analysis between sexes performed with *DMRscaler* showed our algorithm could handle the full range of DMR features present in simulated and real-world samples. Looking at the DMRs between XX and XY individuals, *DMRscaler* was able to identify a small DMR 2.1 kb in length overlapping the autosomal gene *TLE1* that had previously been identified as differentially methylated between the sexes [53], while also consolidating the differential methylation of the X-chromosome into a single DMR 152.13 Mb in length, spanning 98% of the total length of the chromosome.

Additionally, *DMRscaler* provides the means for a hierarchical definition of a DMR that is built through the iterative procedure of merging layers built from increasing window sizes. A deeper analysis of the DMR spanning the X-chromosome showed that gaps in DMRs at lower layers that were consolidated in the upper layers were significantly enriched in genes known to escape X-inactivation, such as *RPS4X* [51, 52], which is concordant with data showing that regions escaping X-inactivation should have a similar epigenetic landscape between sexes [52]. These enrichment results show how *DMRscaler*, in addition to providing an intuitive representation of DMRs, also provides a mechanism for a hierarchical definition of DMRs that can be used to investigate the structure of the methylation landscape across larger epigenomic features. Together these behaviors of intuitive scaling and defining a hierarchical map of DMR features allow *DMRscaler* to be used to achieve greater flexibility and more meaningful interpretation of results in analyses of differential methylation than existing methods.

Finally, given our primary interest in leveraging this method for the smaller sample sizes in rare-disease studies, we tested *DMRscaler* on datasets from patients with rare chromatin modifier syndromes. Specimens that harbor known pathogenic mutations in chromatin modifier genes often display regional changes to epigenetic features, such as DNA methylation state [9, 10]. Our study also explored three syndromes that are caused by pathogenic mutations in genes that directly control histone modifications.

Arboleda-Tham Sydrome (MIM# 616268), also known as KAT6A syndrome, is a genetic syndrome caused by mutations in the Lysine (K) acetyltransferase *KAT6A* characterized by global developmental delay, intellectual disability, speech delay or absence and phenotypes of variable expressivity such as congenital heart defects and gastrointestinal anomalies [7, 58]. KAT6A acetylates histones K3K9, H3K14, and H3K23 [59–61], but the genomic regions affected in Arboleda-Tham syndrome have not been comprehensively studied. Previously, deletion of *KAT6A* in model organisms has identified the *HOX* genes, including the *HOXB* cluster*,* as regulatory targets of KAT6A [60, 62]. Three DMRs identified here were identified by *DMRscaler* spanning multiple genes of the *HOXB* cluster (Fig. 3), which encompass 2 genes (*HOXB3, HOXB4)* found in a *KAT6A* knockout mouse model to have shifted domains of expression resulting in homeotic transformation of the axial skeleton [60]. The ability to highlight the extent of differential methylation beyond a single gene provides further context into the epigenetic change that occurs in Arboleda-Tham syndrome.

One limitation of our study is that cases are generally younger in age than the control groups. Previous studies have identified global hypomethylation as associated with aging [63, 64]. For regions that are largely hypermethylated in Arboleda-Tham Syndrome patients relative to controls, we cannot exclude this as a potential confounding factor in our analysis.

Weaver syndrome and Sotos syndrome are rare overgrowth syndromes that can be difficult to distinguish without sequencing. They are caused by mutations in *EZH2 *[65, 66] and *NSD1* gene [55], respectively. Despite their common clinical phenotype of overgrowth, the regions of the genome that are identified as differentially methylated largely diverge between these two syndromes suggest distinct pathways to a common and complex phenotype. For Weaver Syndrome, *DMRscaler* identified differential methylation over the *HOXA* cluster genes in Weaver syndrome relative to controls. Genome-wide mapping of EZH2 binding domains shows EZH2 binds the *HOXA* cluster [67] and EZH2 overexpression in mantle cell lymphoma has been associated with hypermethylation over the *HOXA* cluster [68]. The key improvement is that rather than highlighting individual genes [9] as differentially methylated, *DMRscaler* is able to demonstrate the modular nature of the genetic regulation by highlighting the non-random spatial relation of these features as a pair of DMRs spanning several genes each.

Additionally, *DMRscaler* identified a novel finding in the neighboring *PCDHA, PCDHB, and PCDHG* clusters as broadly hypermethylated between Sotos syndrome patients relative to controls. The protocadherin family genes are critical in cell–cell adhesion and involved in the complex patterning of neural circuitry [56]. These same genes in the *PCDHGA/B* cluster were also identified as hypermethylated in Down syndrome human cortex relative to control cortex tissue [69]. From these results we can hypothesize that misregulation of the *PCDH* clusters in brain development may contribute to the neurodevelopmental phenotype of Sotos syndrome.

Notably, we observed that between the two overgrowth syndromes, Sotos and Weaver syndrome, the *IGFR2* region including the *INS* and *INS-IGFR2* genes was similarly differentially methylated. Loss of normal imprinting regulation of *IGFR2* has been implicated in another overgrowth syndrome, Beckwith-Wiedemann Syndrome (BWS) [57]. Whether this common difference in DNA methylation proximal to the *IGFR2* locus represents an epigenetic contributor to the overgrowth phenotype or is a consequence of the overgrowth phenotype is worth further investigation.

The majority of real-world methylation data is in the form of reduced representation platforms that query CpGs in sites that are likely to play a role in gene regulation, such as known enhancers and transcriptional start sites. While the distance between the sites are variable on an array, our sex chromsome results demonstrate the ability of our method to call established DMRs that vary dramatically in size on this reduced representation platform. Whole genome bisulfite sequencing (WGBS) offers an alternative to array based technologies for querying DNA methylation that offers more complete coverage of the genome. While WGBS is technically and analytically challenging and remains prohibitively expensive for routine use, DMRscaler is platform agnostic and time efficient on array based data, and so should be readily portable to analysis of WGBS data. Due to the wider availability of array based DNA methylation datasets, particularly for rare disease cohorts, we decided to test DMRscaler on array data and have left validation on WGBS data as a future direction.

## Conclusion

Here we have shown that *DMRscaler* is flexible yet robust in describing the scale of DMR features from the local scale of individual promoters and CpG sites, to the DMR features that represent chromosome level differences in methylation. All of the analyses described were run using a shared parameter set for *DMRscaler*, which highlights the utility to researchers who seek to explore these higher order epigenetic features while also describing the local changes with known biological implication, such as changes in methylation overlapping the promoter of a gene. Importantly, *DMRscaler* serves as a proof of principle. The idea that important epigenetic features exist beyond the scale of a single gene is not new, however, existing methods for DNA methylation analysis do not capture this knowledge. Here *DMRscaler* proves that it is possible to computationally capture this intuition, and in doing so reveal novel biological insights.

## Supplementary Information

**Additional file 1**. Supplemental Figures S1–S25, Supplemental Tables S1, S7, S8, S10–S12.

**Additional file 2. Table S2:** Differential sex analysis DMRs.

**Additional file 3. Table S3:** Arboleda-Tham syndrome DMRs

**Additional file 4. Table S4:** Weaver syndrome DMRs

**Additional file 5. Table S5:** Sotos syndrome DMRs

**Additional file 6 Table S6:** Syndrome overlaps

**Additional file 7 Table S9:** Simulation results

## Data Availability

The code for the simulation analysis is available at https://github.com/leroybondhus/dmrscaler_simulation. The code for the real data analysis is available at https://github.com/leroybondhus/dmrscaler_real_data. Dataset from Arboleda-Tham Syndrome can be found with GEO accession: GSE210484 at https://www.ncbi.nlm.nih.gov/geo/query/acc.cgi?acc=GSE210484. Datasets from Sotos and Weaver Syndrome can be found with GEO accession: GSE74432 at https://www.ncbi.nlm.nih.gov/geo/query/acc.cgi?acc=GSE74432. Methods used for comparison can be found at DMRcate [46]: https://www.bioconductor.org/packages/release/bioc/html/DMRcate.html, bumphunter[44]: https://www.bioconductor.org/packages/release/bioc/html/bumphunter.html, comb-p[45]: https://github.com/brentp/combined-pvalues. Packages used for visualization of data Hilbert, Curves [71]: https://www.bioconductor.org/packages/release/bioc/html/HilbertCurve.html, Genomic Range Plots [72]: http://bioconductor.org/packages/release/bioc/html/Gviz.html, Network Plots [73]: https://cran.r-project.org/web/packages/networkD3/index.html. **Availability and requirements** Project Name: DMRscaler. Project home page: https://github.com/leroybondhus/DMRscaler. Operating system(s): Platform independent. Programming language: R [70]. Other requirements: R version 4.1.0 or higher. License: MIT. Any restrictions to use by non-academics: None.

## Abbreviations

TF: Transcription factor
PRD: Polycomb repressive domain
TAD: Topologically associated domain
DNAme: DNA methylation
CpG: Cytosine guinine dinucleotide
VUS: Variant of unknown significance
DMR: Differentially methylated region
FDR: False discovery rate
QC: Quality control
TP: True positive
FN: False negative
FP: False positive
AUCPR: Area under precision recall curve
