# Supplementary material for: DMRscaler: a scale-aware method to identify regions of differential DNA methylation spanning basepair to multi-megabase features
Source: BMC Bioinformatics. 2022 Sep 5;23:364. doi: 10.1186/s12859-022-04899-1 (PMC9447346; doi:10.1186/s12859-022-04899-1)
Supplement: Supplementary file 1 — Additional file 1. Supplemental Figures S1–S25, Supplemental Tables S1, S7, S8, S10–S12. [file 12859_2022_4899_MOESM1_ESM.pdf]

Supplemental Figures for:

DMRscaler: A Scale-Aware Method to Identify Regions of Differential DNA Methylation Spanning Basepair to Multi-Megabase Features

Leroy Bondhus[1,3,4], Angela Wei[1,2,3,4], Valerie A.Arboleda[1,2,3,4,5,6*]

1 Department of Human Genetics, David Geffen School of Medicine, UCLA, Los Angeles, CA 90095

2 Bioinformatics Interdepartmental PhD Program, David Geffen School of Medicine, UCLA, Los Angeles, CA 90095

3 Department of Pathology and Laboratory Medicine, David Geffen School of Medicine, UCLA, Los Angeles, CA 90095

4 Department of Computational Medicine, David Geffen School of Medicine, UCLA, Los Angeles, CA 90095

5 Molecular Biology Institute, UCLA, Los Angeles, CA 90095

6 Jonsson Comprehensive Cancer Center, UCLA, Los Angeles, CA, 90095, USA, Los Angeles, CA 90095

*Corresponding Author

Valerie A. Arboleda, MD PhD
615 Charles E. Young Drive South
Los Angeles, CA 90095
310-983-3568
varboleda@mednet.ucla.edu

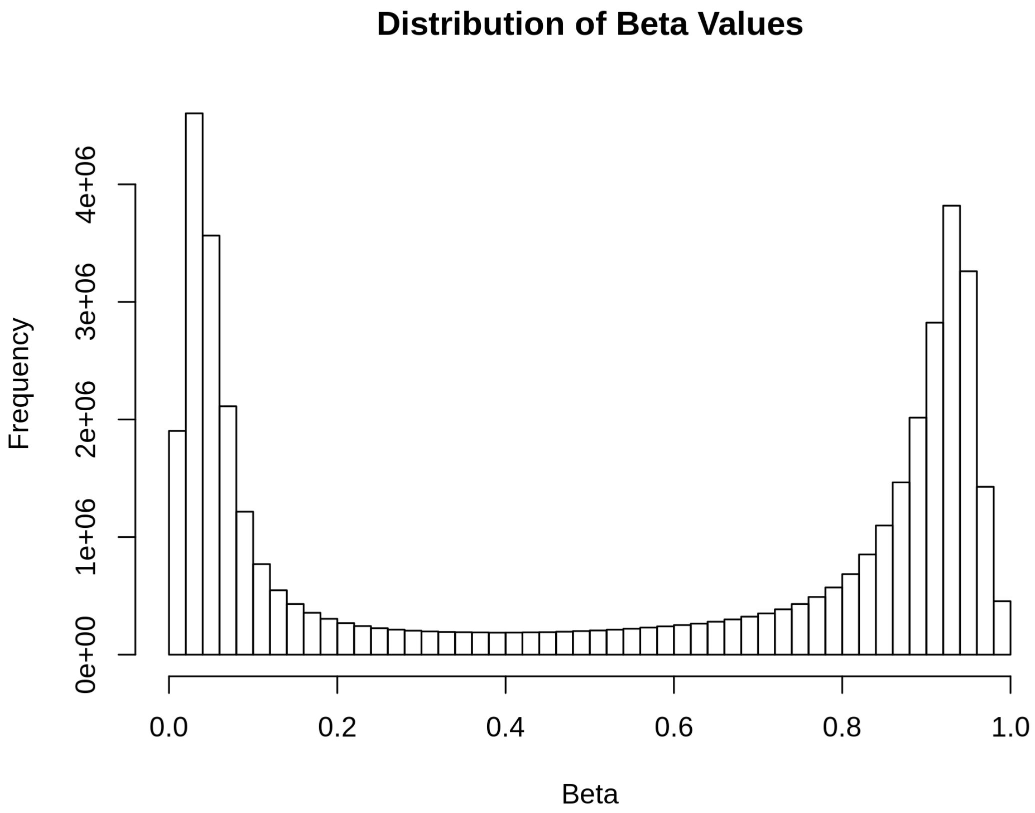

Figure S1. Beta Distribution of methylation data from Illumina Infinium Human Methylation450 Bead Chip 450 array. Beta value is the proportion of methylation at each CG site.

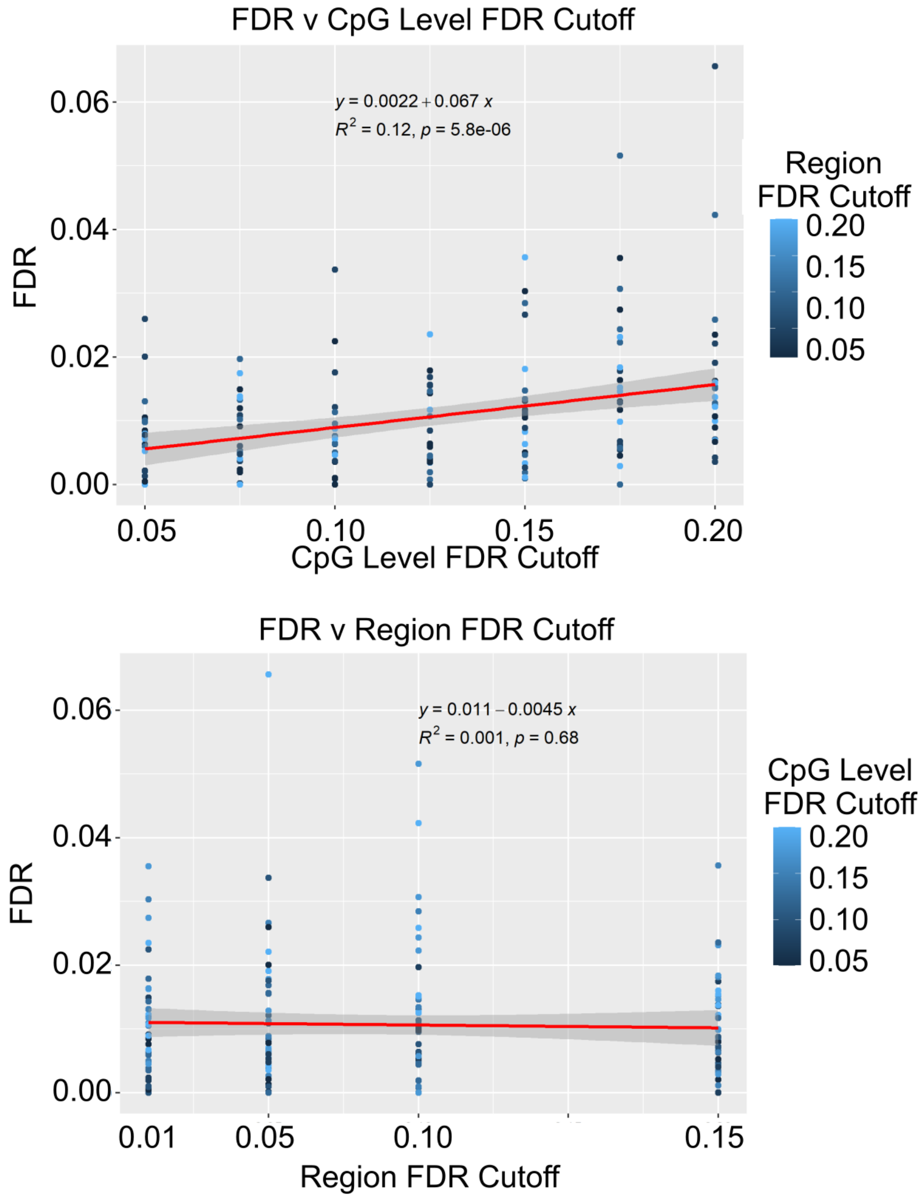

Figure S2: Test of FDR control from the CpG level FDR cutoff which is the p-value at which the set FDR is achieved based on permutation, and the region FDR cutoff, which is the FDR level set and determined by the Benjamini-Yekutieli procedure.

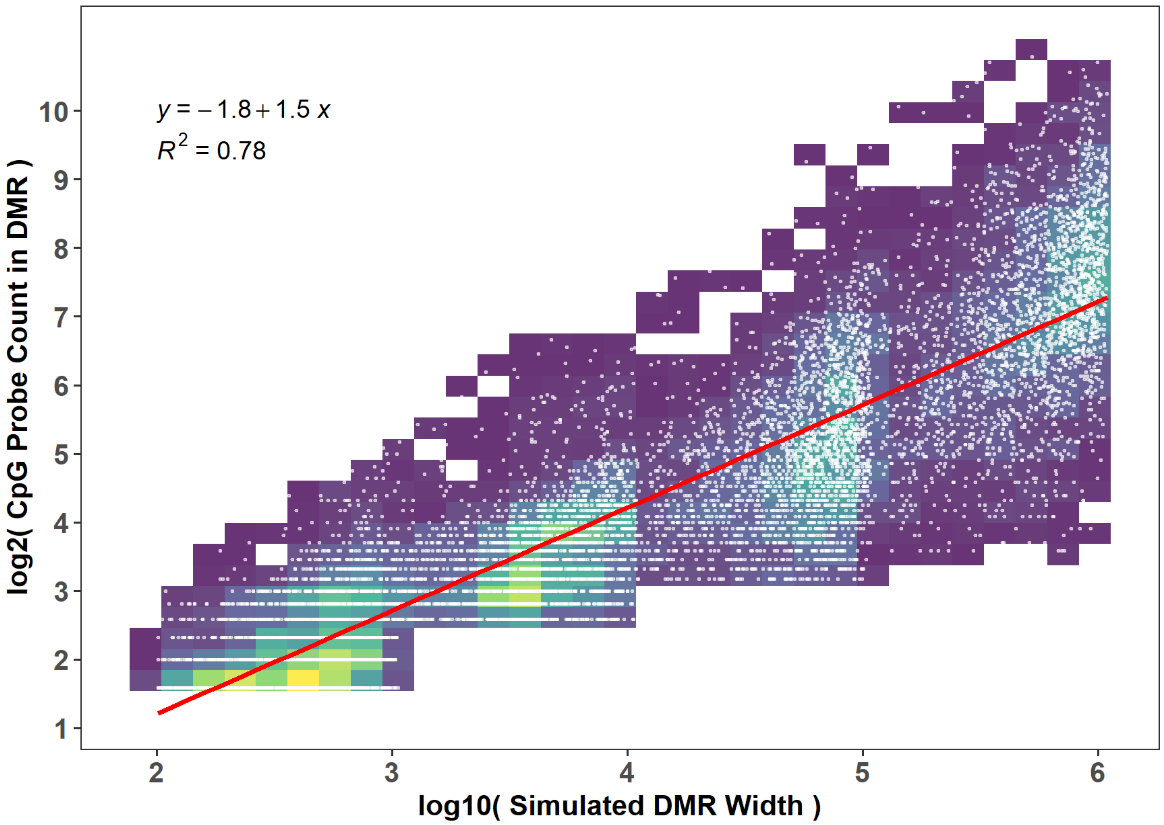

Figure S3: Simulated DMR width vs number of CpG probes. Plot shows distribution of CpG probes per simulated DMR against the simulated DMR width across all simulations run. Regression line and correlation included in upper, left corner of plot.

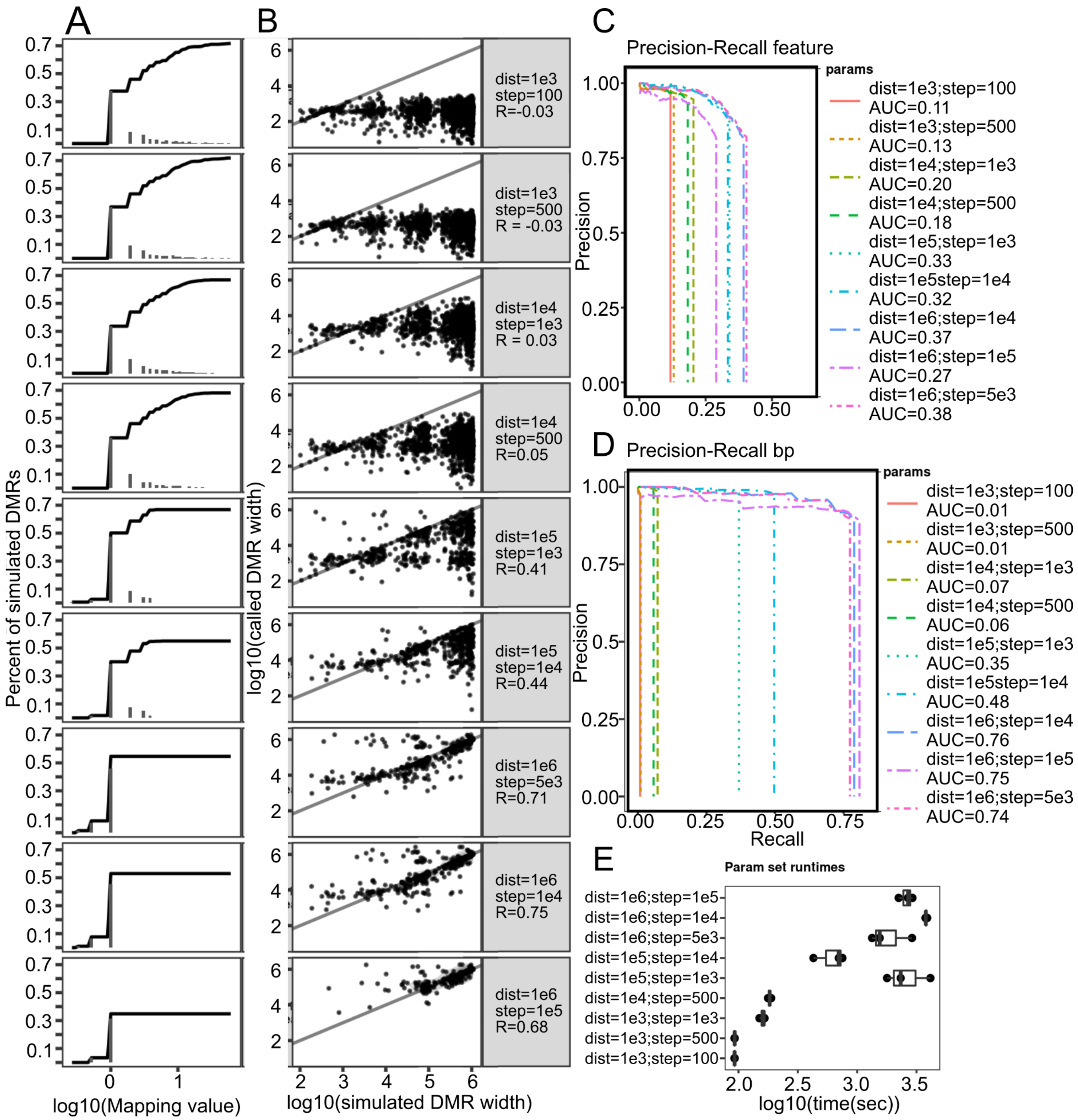

Figure S4: comb-p parameter testing: **(A)** Mapping Values plots. Log values > 0 imply multiple DMRs called per simulated DMR. Value < 0 imply multiple simulated DMRs overlap single called DMR. Value = 0 implies one DMR called per DMR simulated. The plotted line indicates the cumulative proportion of simulated DMRs up to the given mapping value. **(B)** Simulated DMR Widths v Called DMR Widths plotted on log10 scale.**(C)** Feature level precision-recall curves, see methods for details on calculation. **(D)** basepair level precision recall curves **(E)** time for each parameter set run on the simulated dataset across 3 runs.

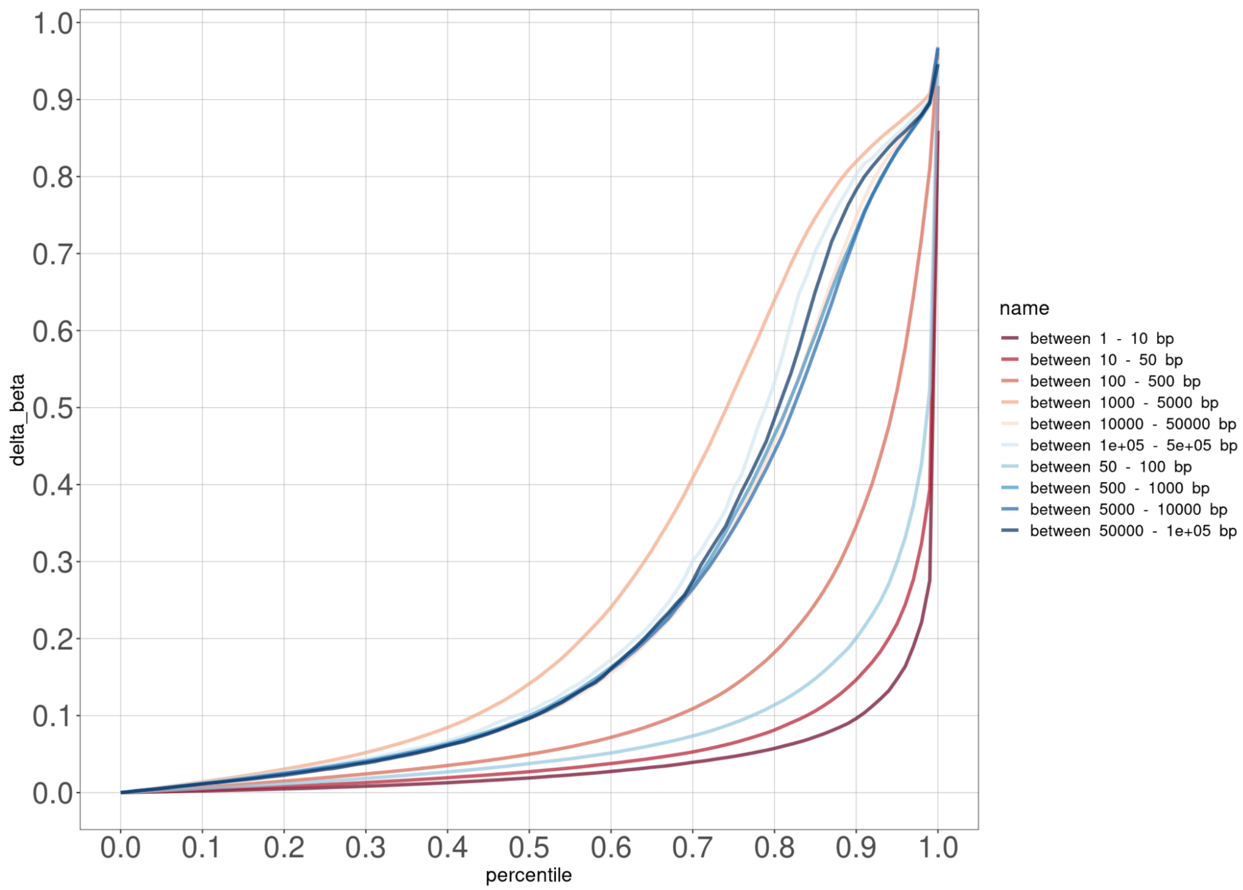

Figure S5: Cumulative Probability of difference in beta values between neighboring CG methylation. Y axis is the difference in Beta between neighboring CGs (CG_i and CG_i+1). E.g. 70% of adjacent measured CGs that are between 1000-5000 bp apart have a difference in beta value less than 0.40, said another way, 30% have a difference in beta greater than 0.4.

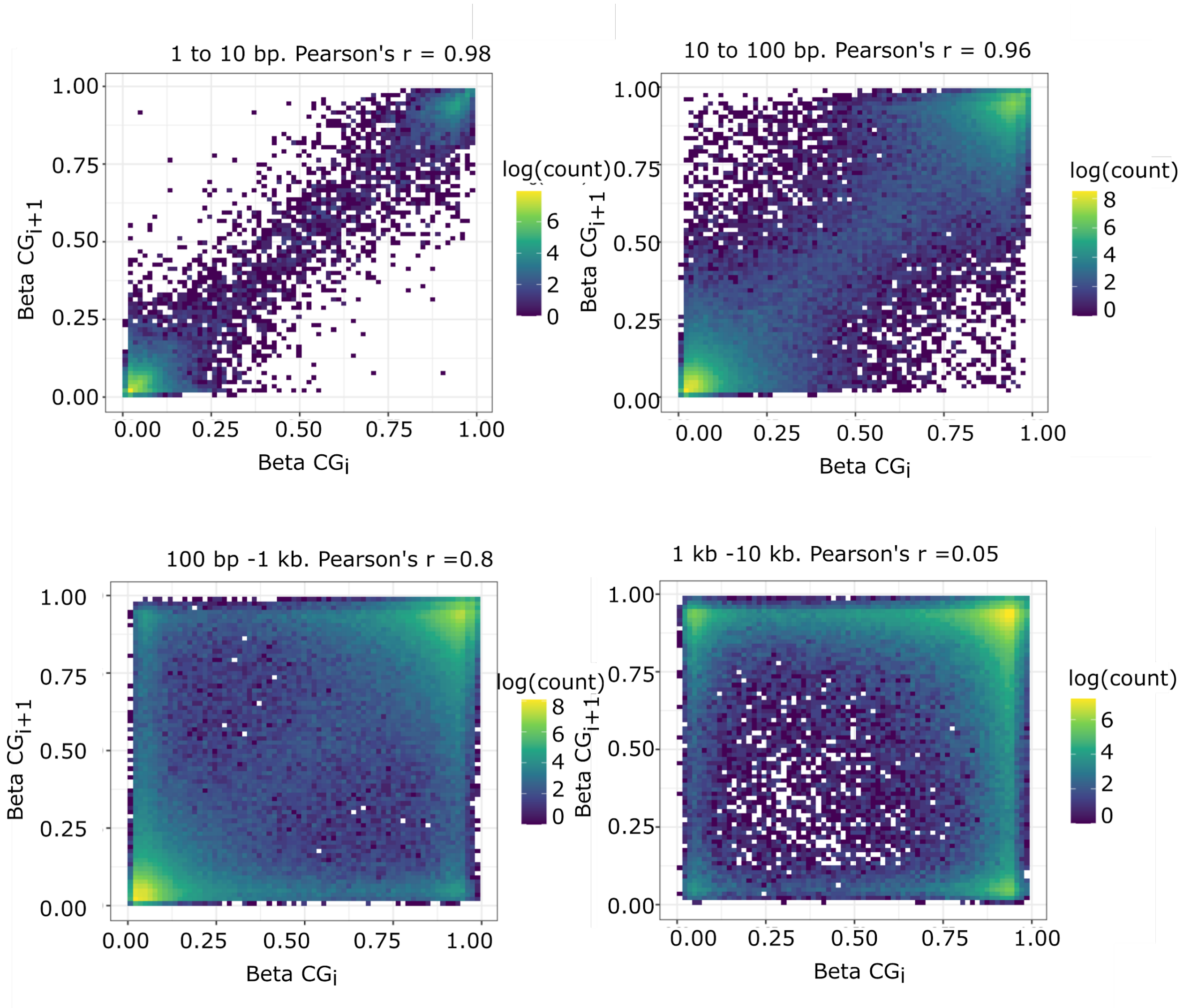

Figure S6: Neighboring CG methylation correlation. Plotted is beta value from 0-1, for CG_i on the x-axis and CG_i+1 on the y-axis. Neighboring CGs 1-10 bp apart and 10-100 bp apart have strong, but not perfect correlation (Pearson's r = 0.98, 0.96), those 100-1000 bp apart have modest correlation (Pearson's r = 0.8). At 1-10kb distance the correlation becomes weak (Pearson's r = 0.05).

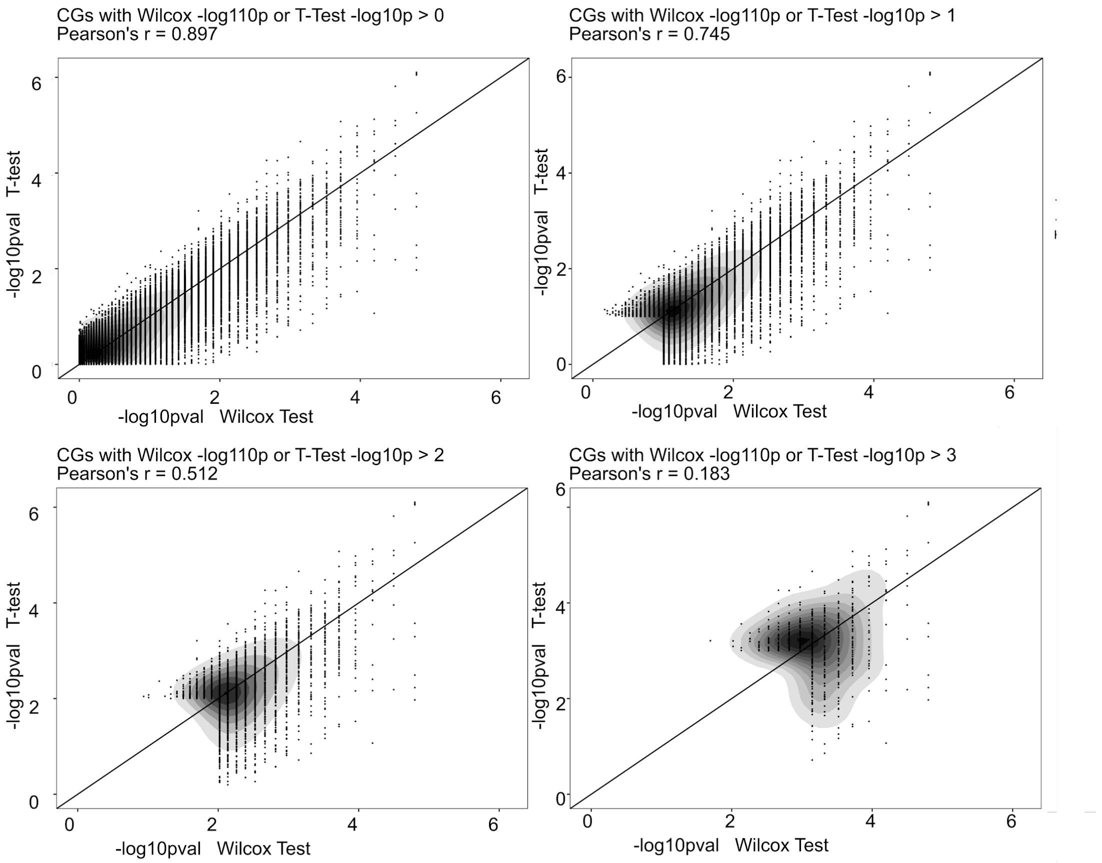

Figure S7: Wilcox - T-Test pearson's correlation (pearson's r) for each CG. From Arboleda-Tham Syndrome data, case-control labels determine group.

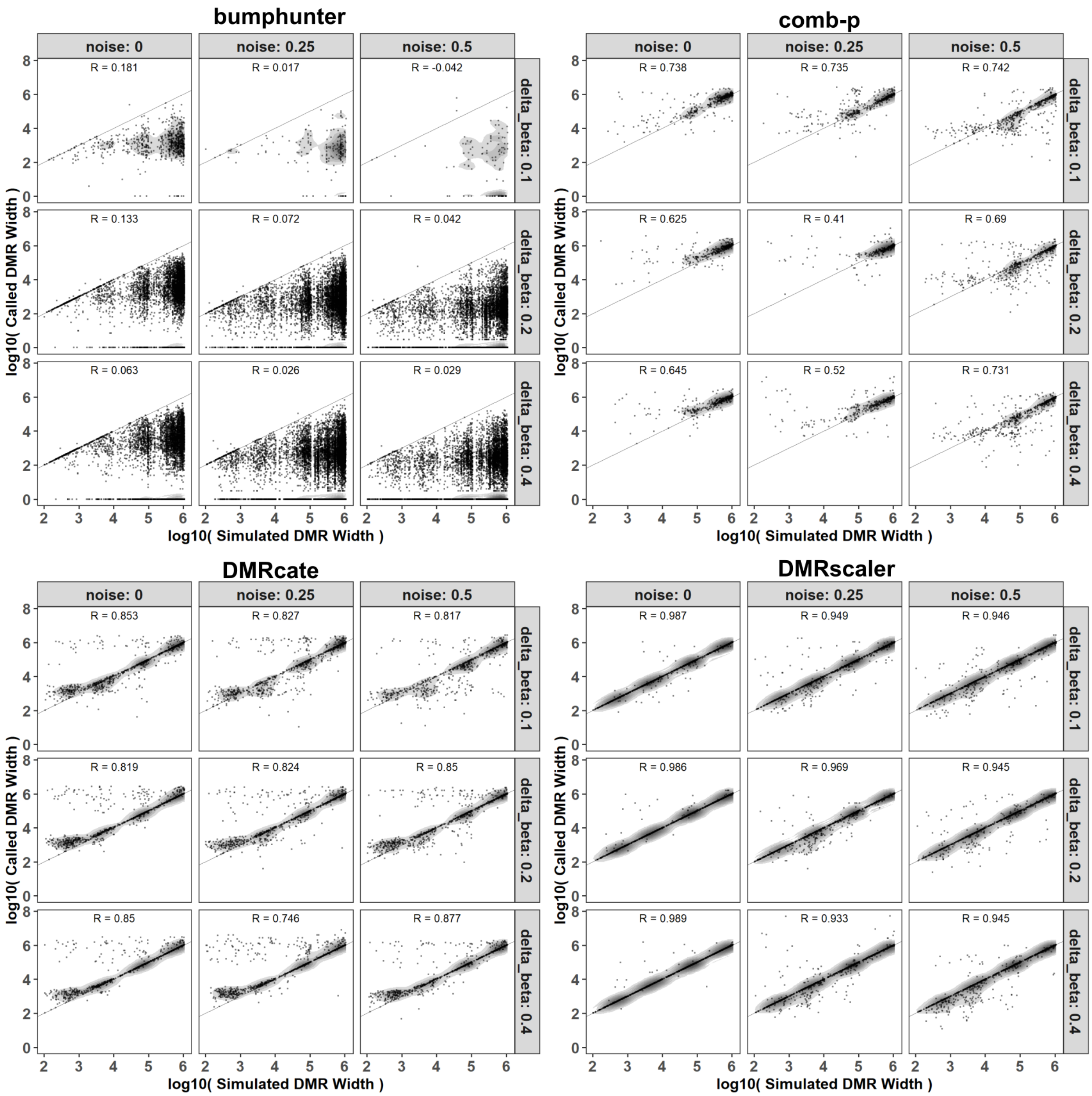

Figure S8: Simulated vs called DMR width for varied noise and delta_beta parameters. Simulated DMR Widths v Called DMR Widths plotted on log10 scale. Pairs are formed between simulated and called DMRs if there is any overlap between the two.

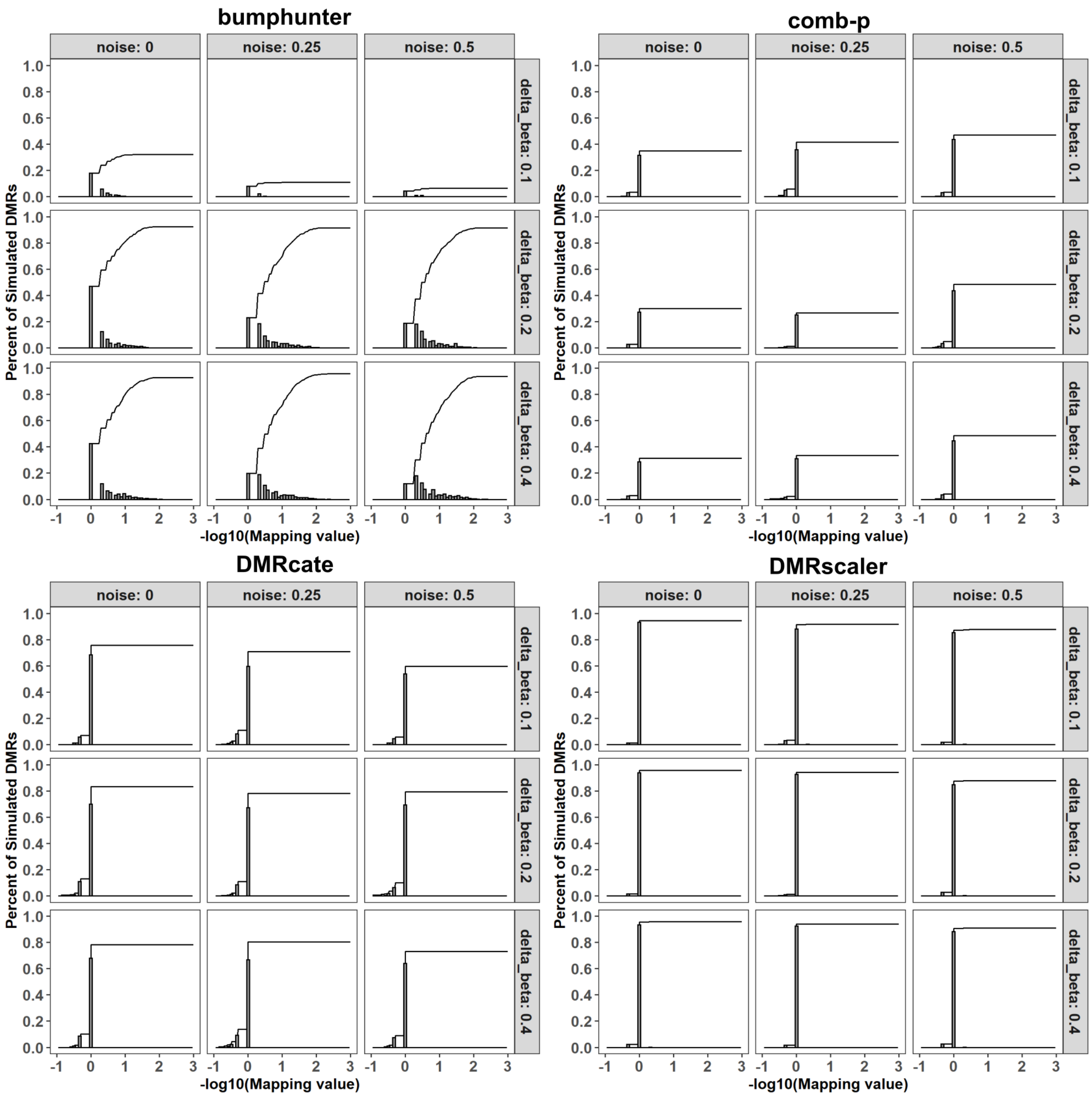

Figure S9: Mapping Values plots for varied noise and delta_beta parameters. The mapping value is calculated for each simulated DMR and is either the inverse of the number of simulated DMRs sharing an overlapping called DMR or else it is the number of called DMRs overlapping the given simulated DMR, whichever is more extreme. Log values > 0 imply multiple DMRs called per simulated DMR. Value < 0 imply multiple simulated DMRs overlap single called DMR. Value = 0 implies one DMR called per DMR simulated. The plotted line indicates the cumulative proportion of simulated DMRs up to the given mapping value.

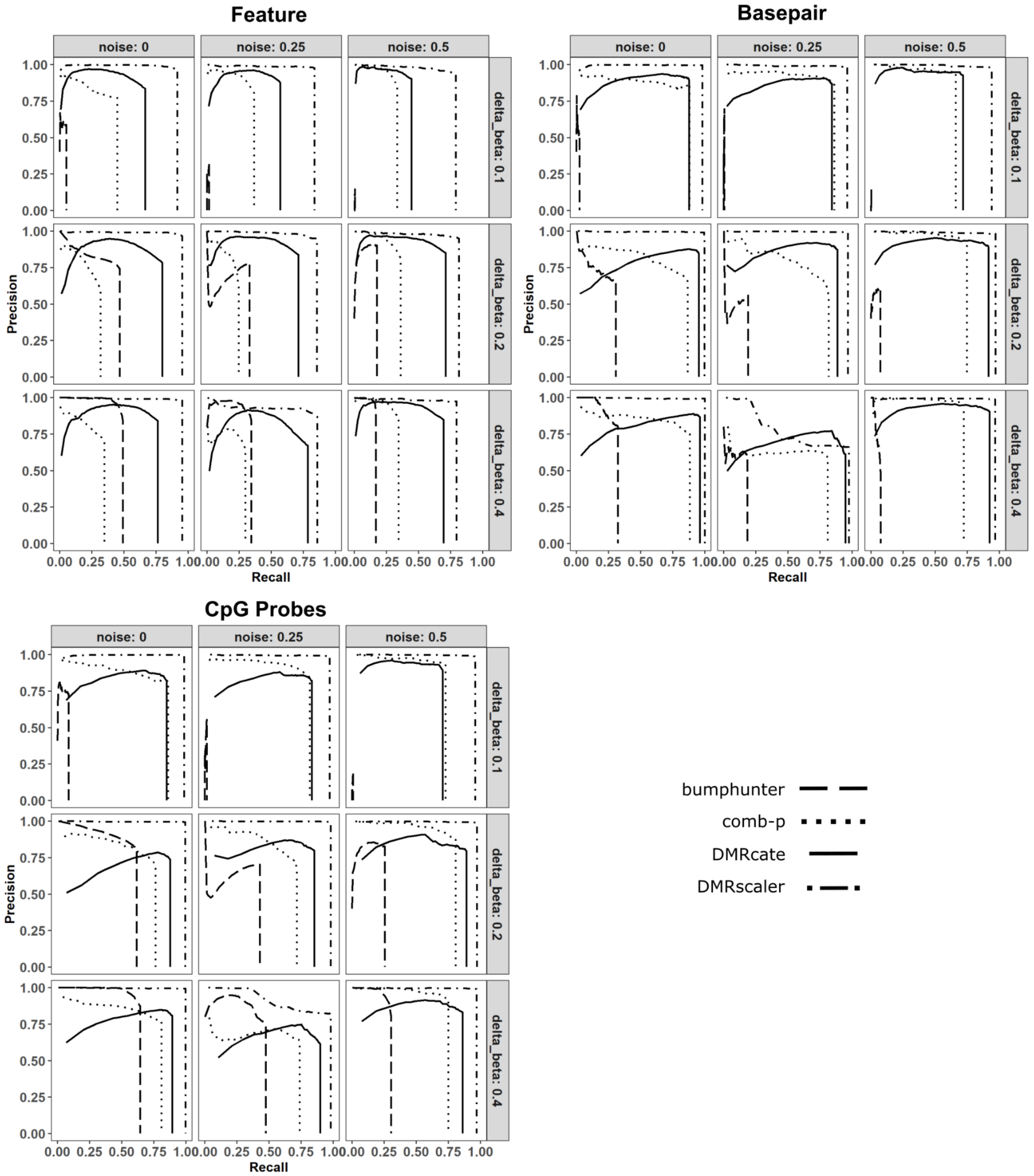

Figure S10: Precision and recall curves with varied simulation parameters for delta_beta and noise for each method, see methods for details on calculation.

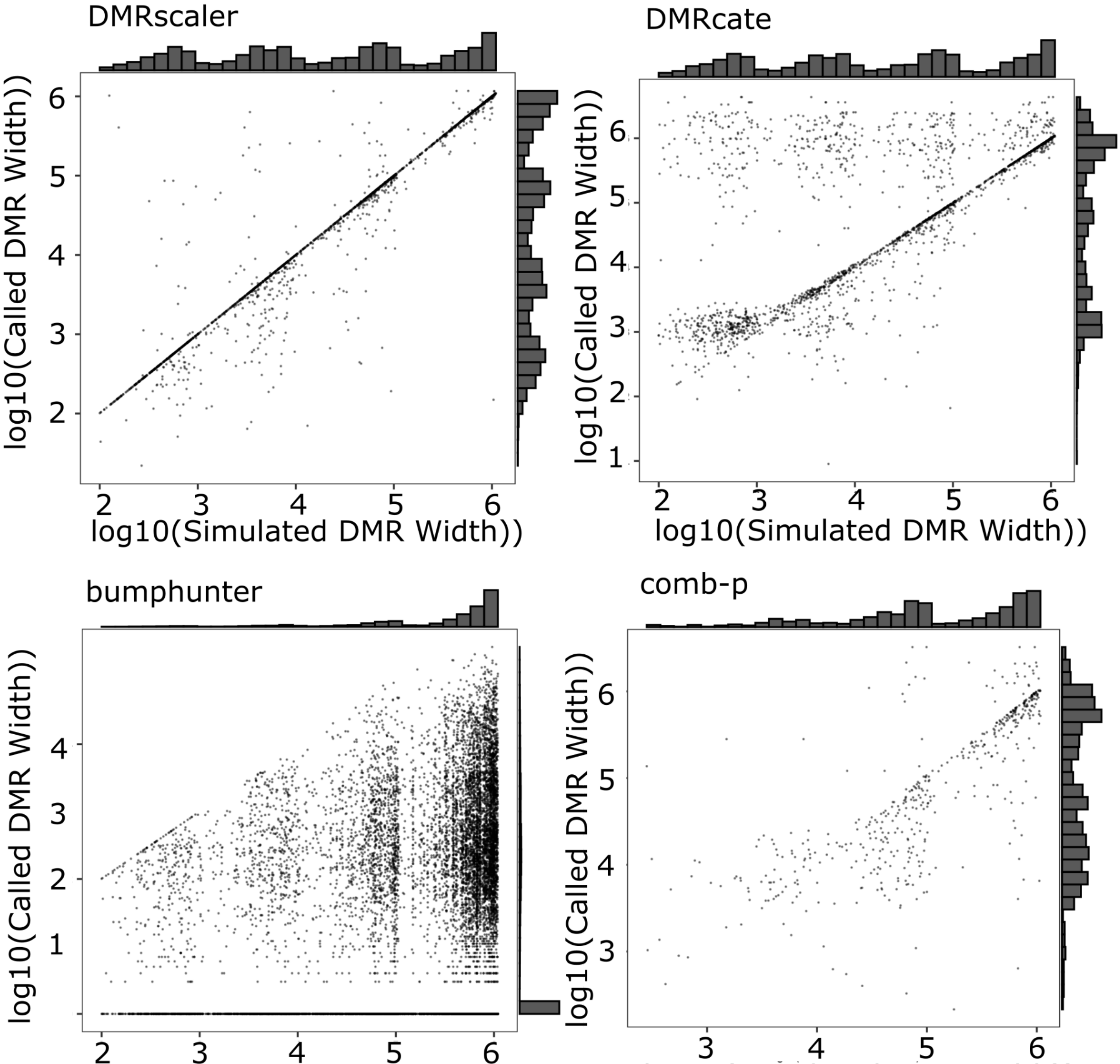

Figure S11. Simulated vs Called Widths with marginal density plots for noise = 50% and delta_beta = 0.2

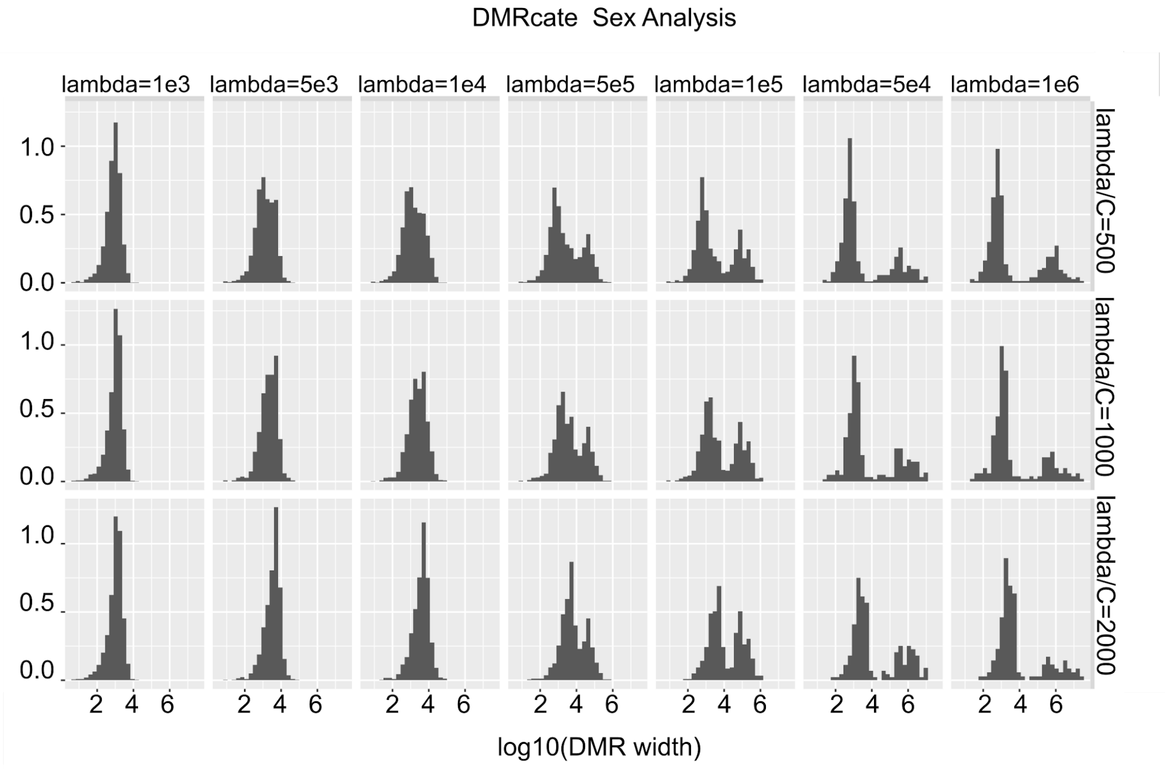

Figure S12: Testing variable lambda and C parameters on output from sex analysis for DMRcate. Only showing autosomal DMRs.DMRs called using a subset consisting of 8 XY and 8 XX controls.

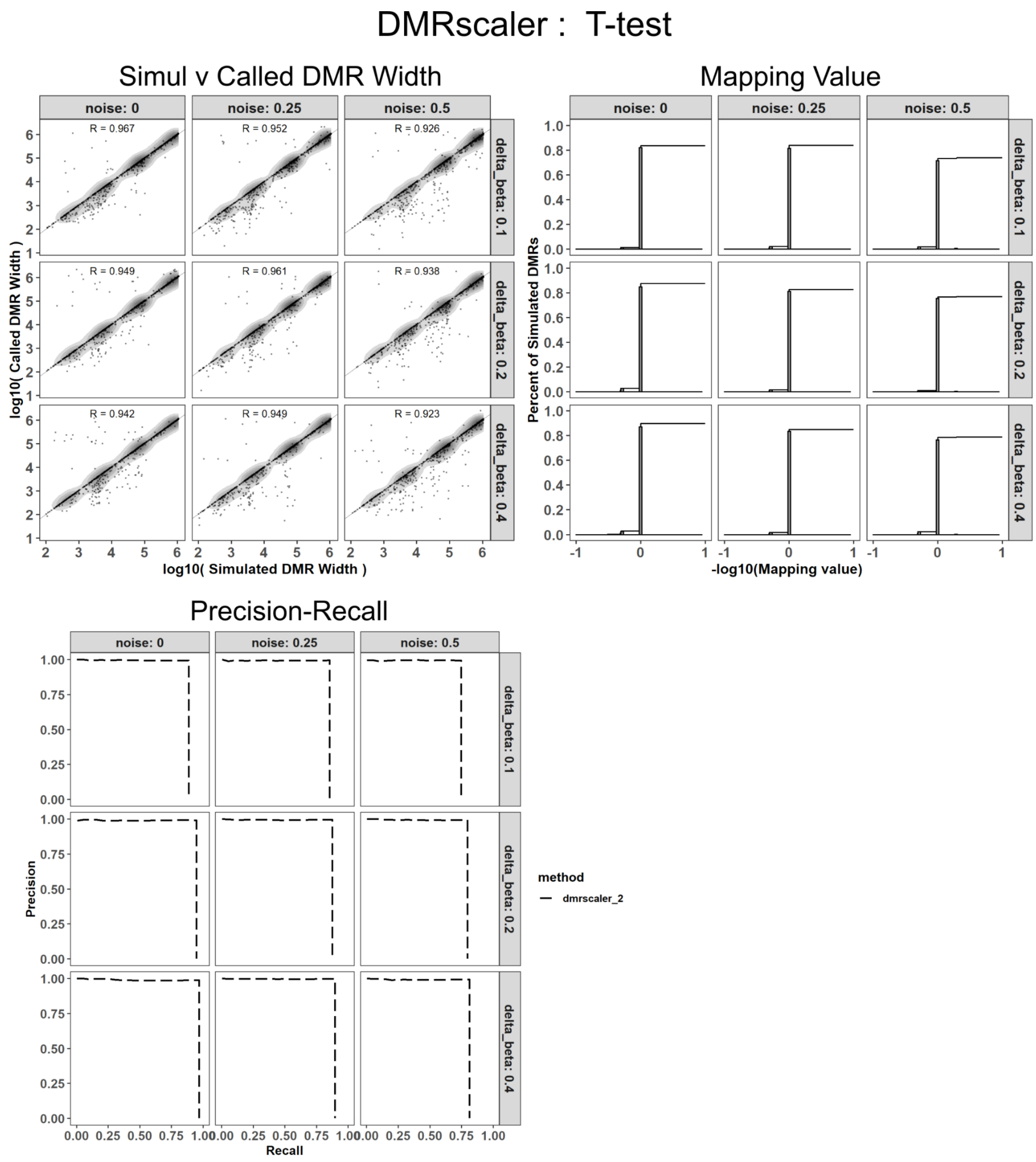

Figure S13: Simulation result for *DMRscaler* run on T-test derived p-values. Top left: Simulated vs called DMR width for varied noise and delta_beta parameters. Pairs are formed between simulated and called DMRs if there is any overlap between the two. Top right: The mapping value for each simulated DMR is either the inverse of the number of simulated DMRs sharing an overlapping called DMR or the number of called DMRs overlapping the given simulated DMR, whichever is more extreme. Bottom: Precision and recall curves with varied simulation parameters for delta_beta and noise for each method, see methods for details on calculation.

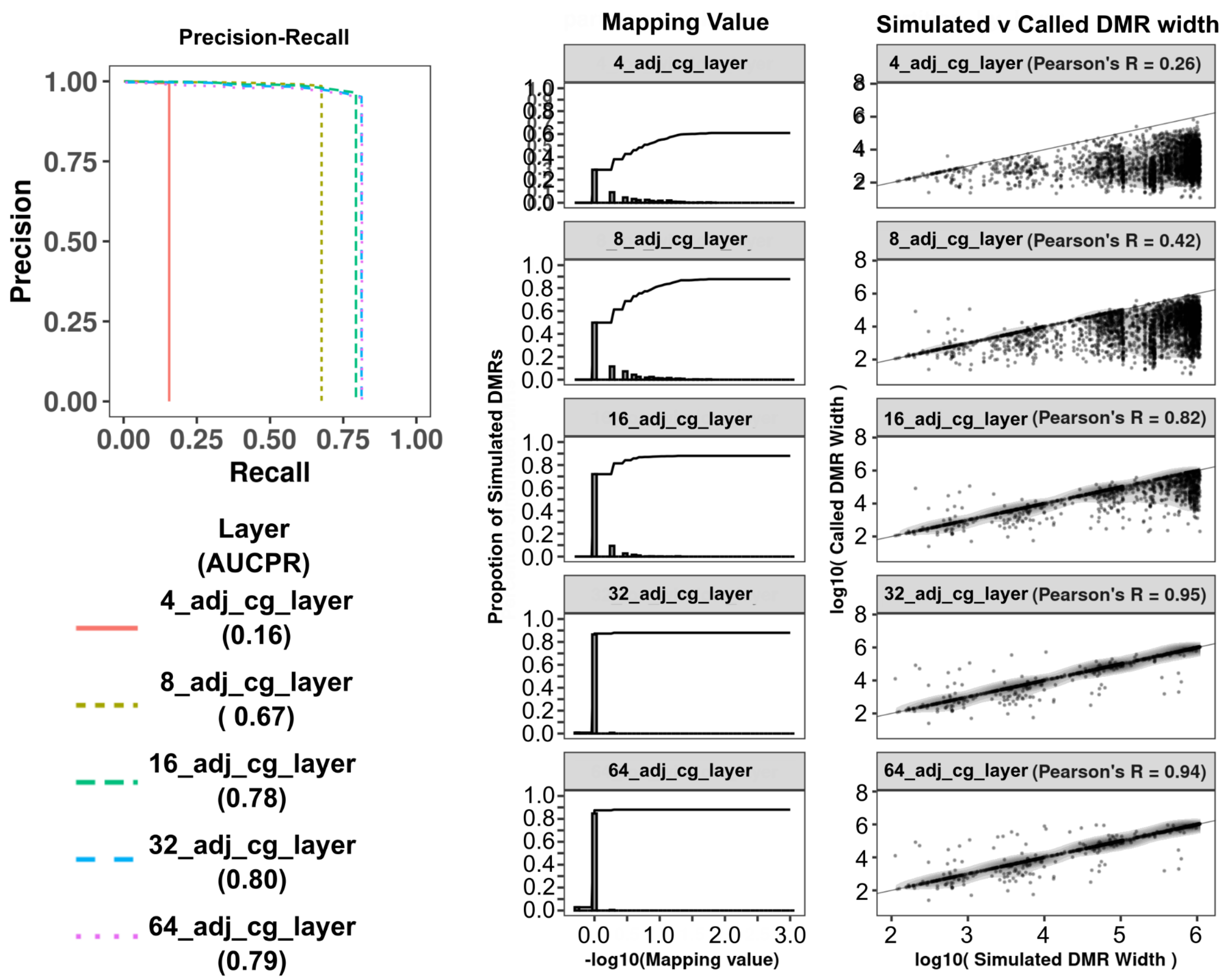

Figure S14: Performance measured at each layer of DMRscaler algorithm Top left: Precision and recall curves for each layer, see methods for details on calculation. Middle: The mapping value for each simulated DMR is either the inverse of the number of simulated DMRs sharing an overlapping called DMR or the number of called DMRs overlapping the given simulated DMR, whichever is more extreme. Right: Simulated vs called DMR width. Pairs are formed between simulated and called DMRs if there is any overlap between the two.

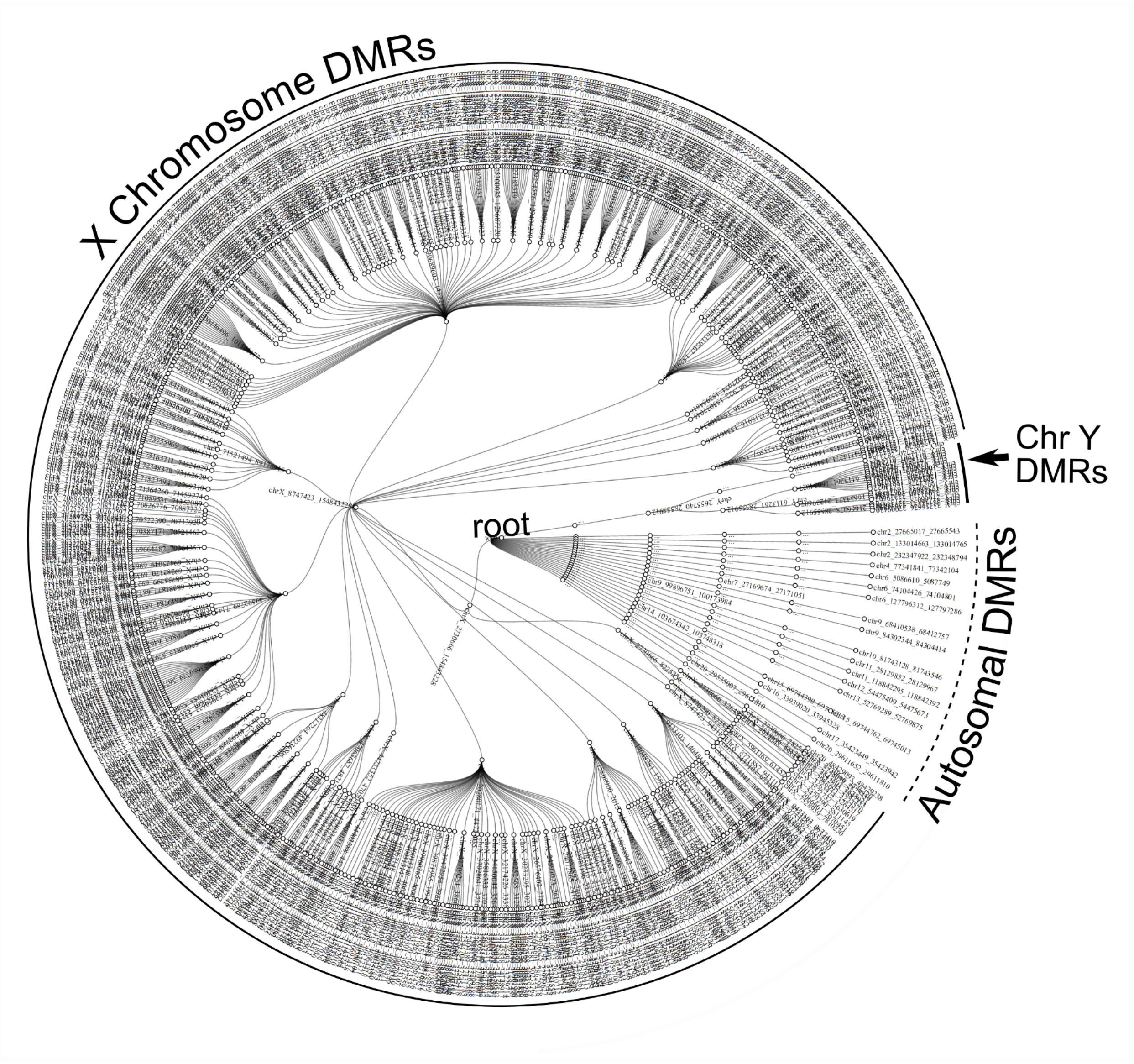

Figure S15. Radial Network Showing hierarchical structure of DMRs called across all layers of the DMRscaler algorithm from layer 1 (4 adjacent CG windows) at the edges nodes to layer 5 (64 CG windows) in the inner ring. Note, all are connected to the virtual root node which is only used for plotting purposes here. Each node is an individual DMR called. Coordinates for each DMR are printed at the earliest layer where that DMR appears. Unlabelled nodes are those that did not change from the previous layer.

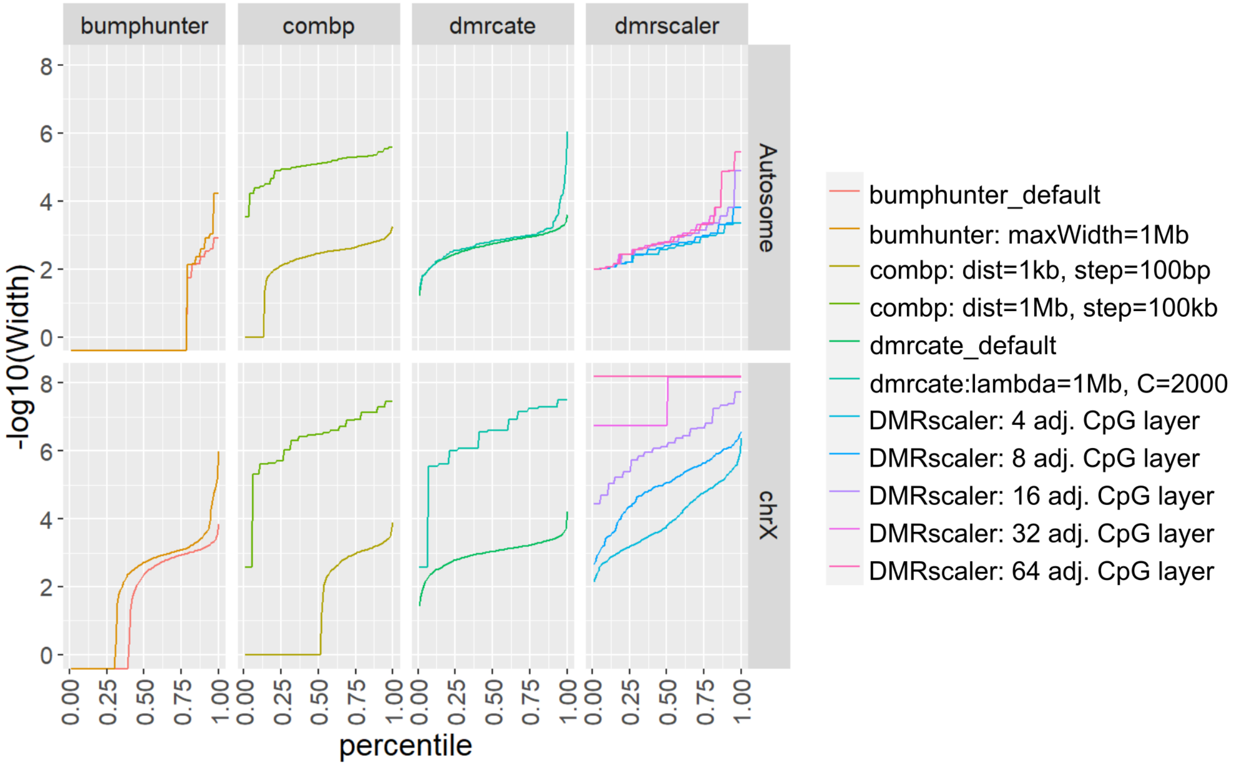

Figure S16: Sex analysis DMR width percentile plot. DMRs Called by each method for sex analysis ordered by dmr width.

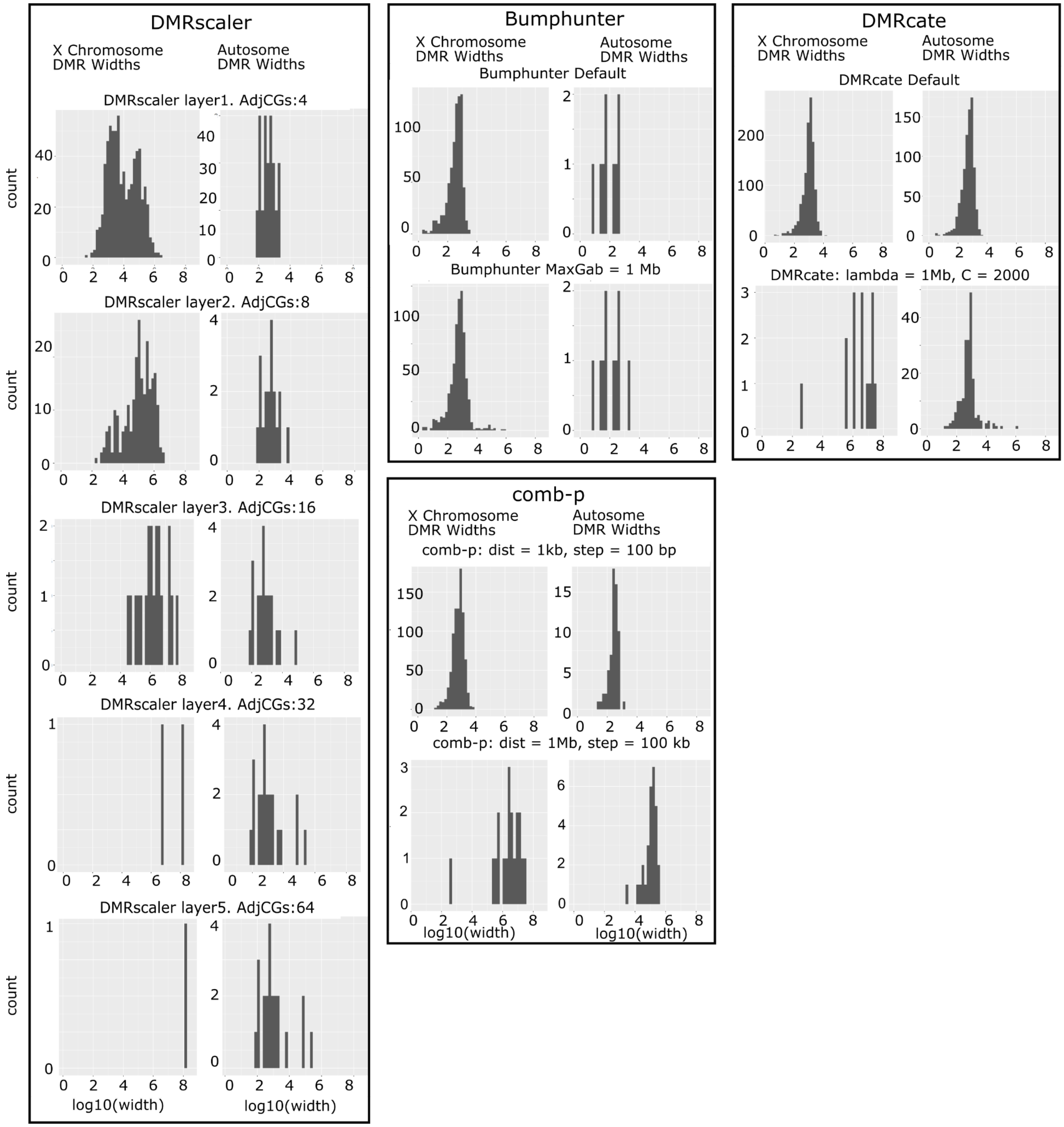

Figure S17 : Distribution of DMR widths for each method called in XX vs XY sex chromosome analysis.

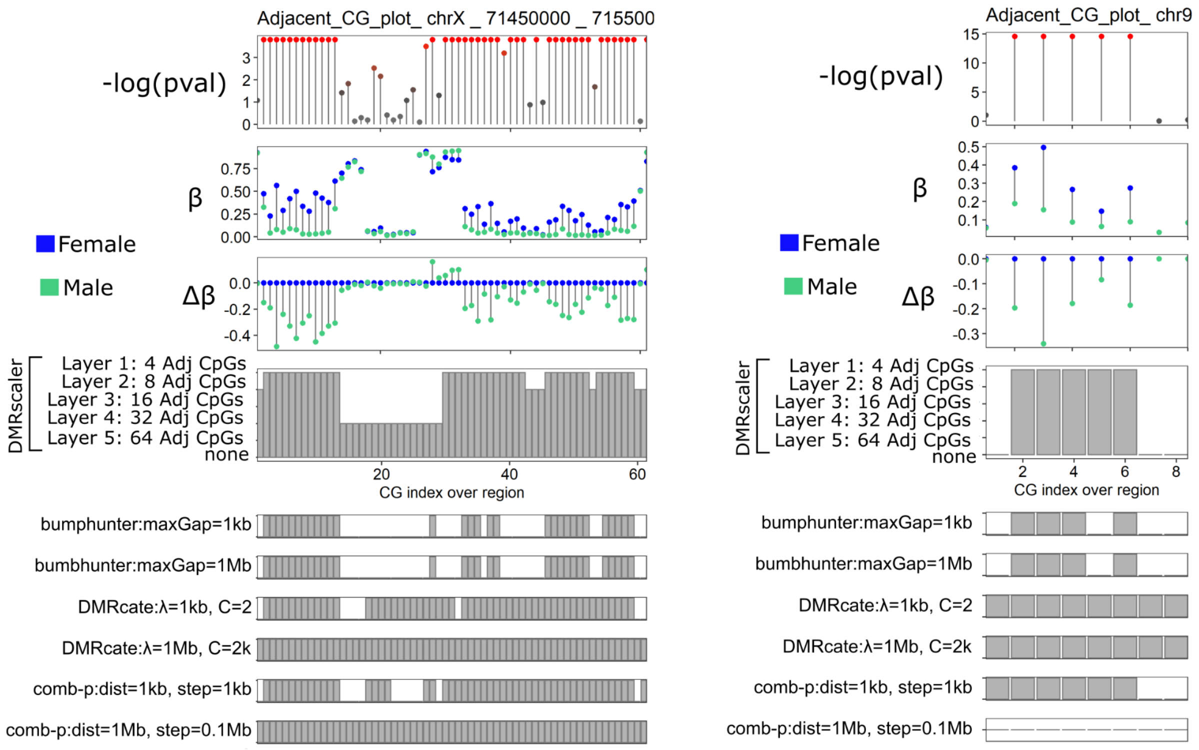

Figure S18: Sex analysis. Supplement to Figure 3C (left), 3D (right). Adjacency plot of CGs overlapping specified regions. Top panel is significance at individual CG level. Beta plot shows mean beta value for each group. Delta beta below shows mean beta value for each group relative to female values. Bottom plot shows in grey bars which layer or method a DMR was called in and from each competing method.

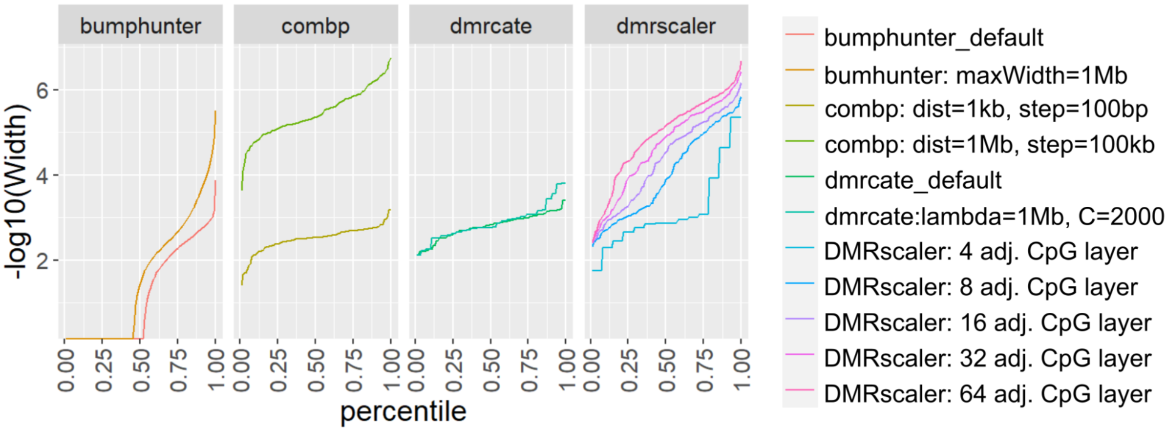

Figure S19: Arboleda-Tham Syndrome analysis DMR width percentile plot. DMRs Called by each method for Arboleda-Tham Syndrome analysis ordered by dmr width.

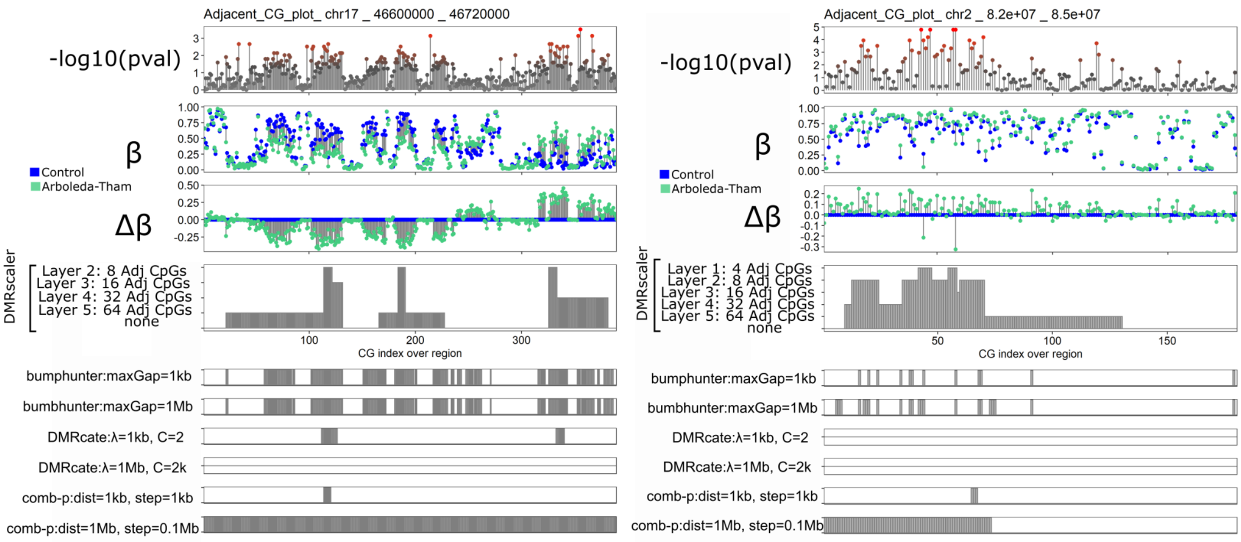

Figure S20: Arboleda-Tham analysis. Supplement to Figure 4B (left), 4D (right). Adjacency plot of CGs overlapping specified regions. Top panel is significance at individual CpG level. Beta plot shows mean beta value for each group. Delta beta below shows mean beta value for each group relative to female values. Bottom plot shows in grey bars which layer or method a DMR was called in and from each competing method.

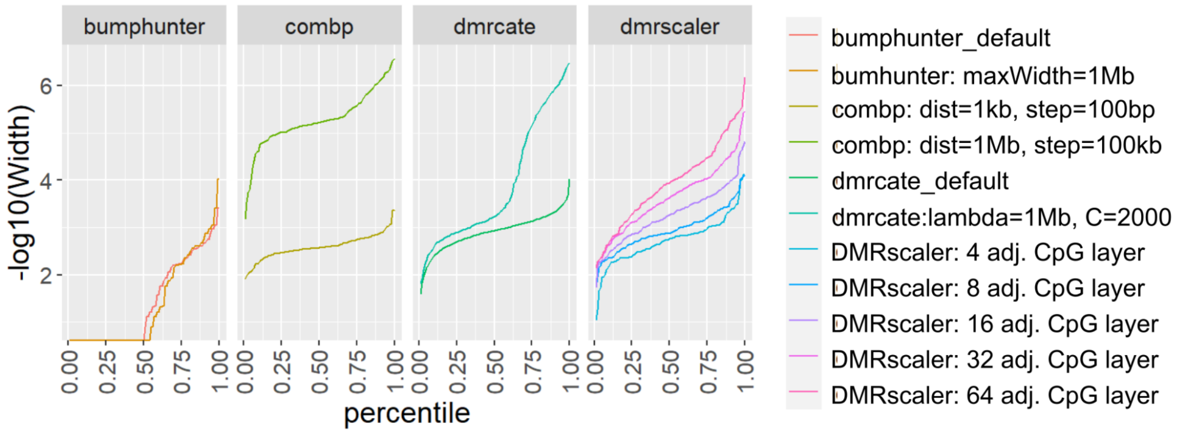

FIGURE S21: Weaver syndrome analysis DMR width percentile plot. DMRs Called by each method for Weaver syndrome analysis ordered by dmr width.

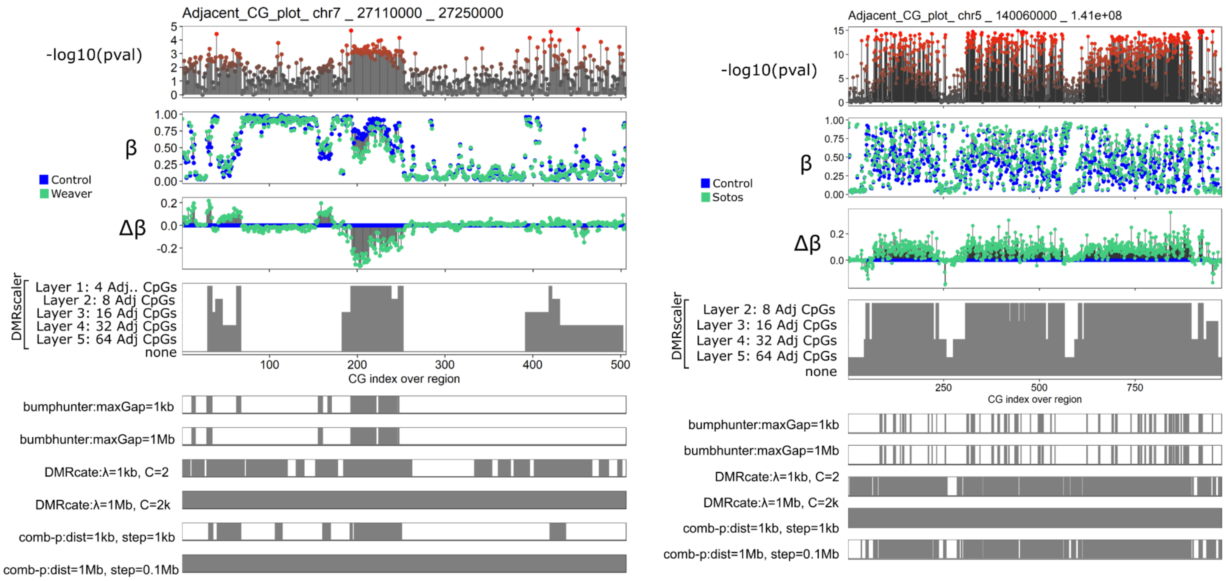

Figure S22: Weaver (left) and Sotos (right) analyses. Supplement to Figure 5B (left), 5D (right). Adjacency plot of CGs overlapping specified regions. Top panel is significance at individual CG level. Beta plot shows mean beta value for each group. Delta beta below shows mean beta value for each group relative to female values. Bottom plot shows in grey bars which layer or method a DMR was called in and from each competing method..

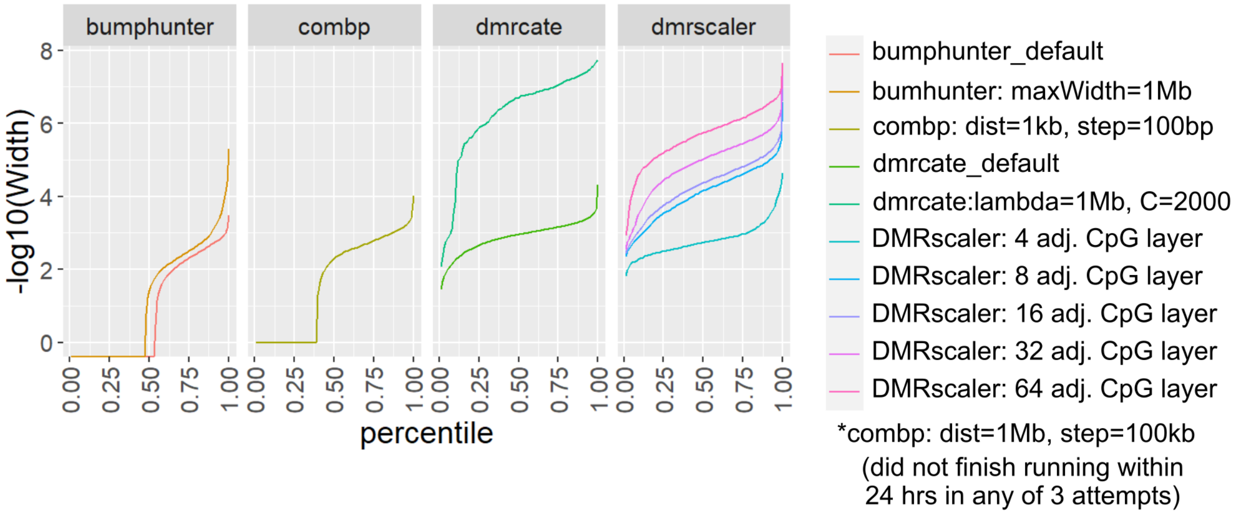

FIGURE S23: Sotos syndrome analysis DMR width percentile plot. DMRs Called by each method for Sotos syndrome analysis ordered by dmr width.

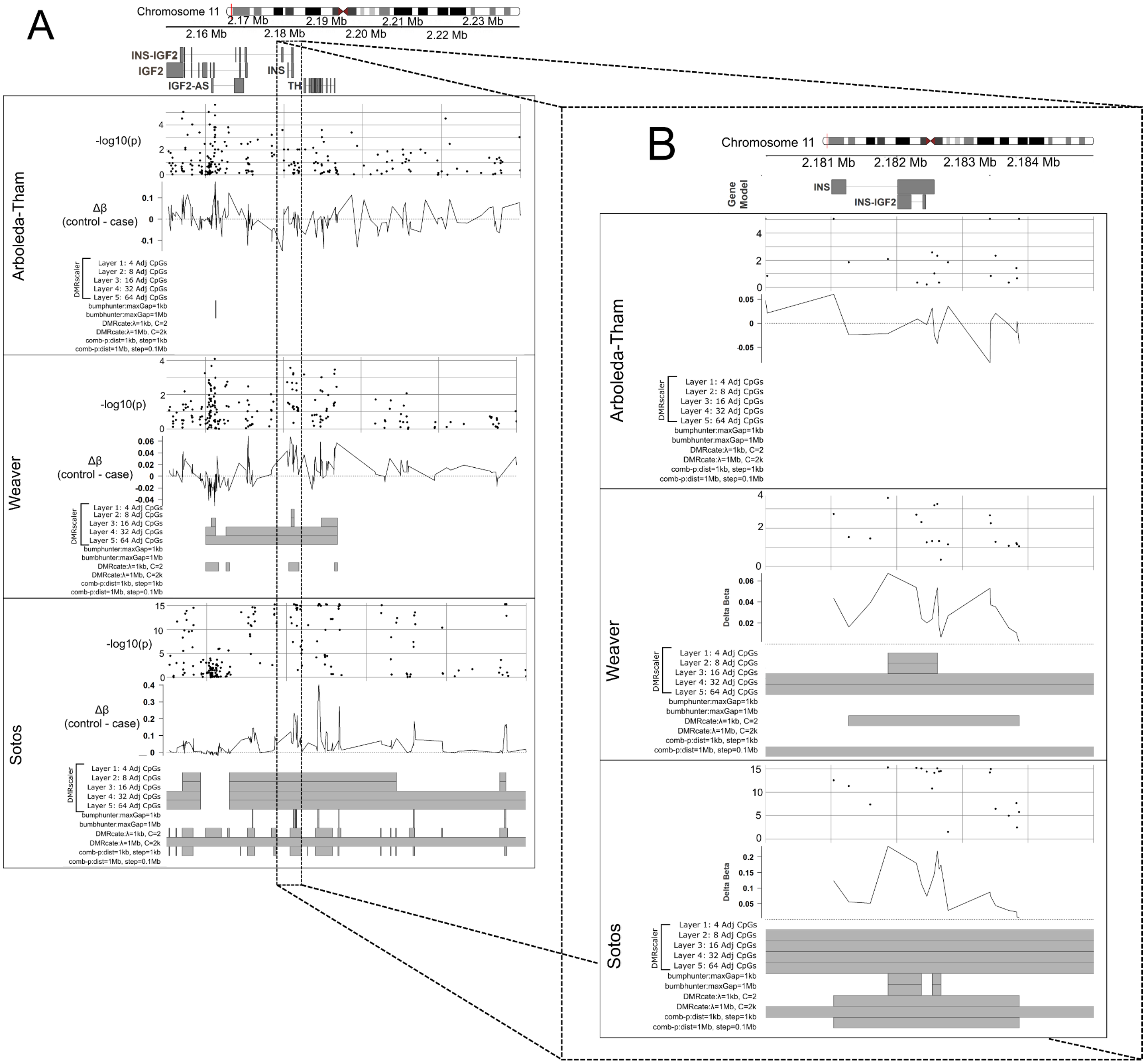

Figure S24: *INS, IGF2, INS-IGF2* region. Overlap between the Sotos and Weaver Syndromes, two overgrowth syndromes, identified a region proximal to and overlapping *INS, IGF2*, and *INS-IGF2*. Top track shows -log10(p) significance value for the dataset specified at left, next track shows Δβ, where $\Delta\beta = \beta_{Case} - \beta_{Control}$. Below the gene model track is the DMR track, highlighting the regions called as a DMR at each result layer from DMRscaler and from each competing method.

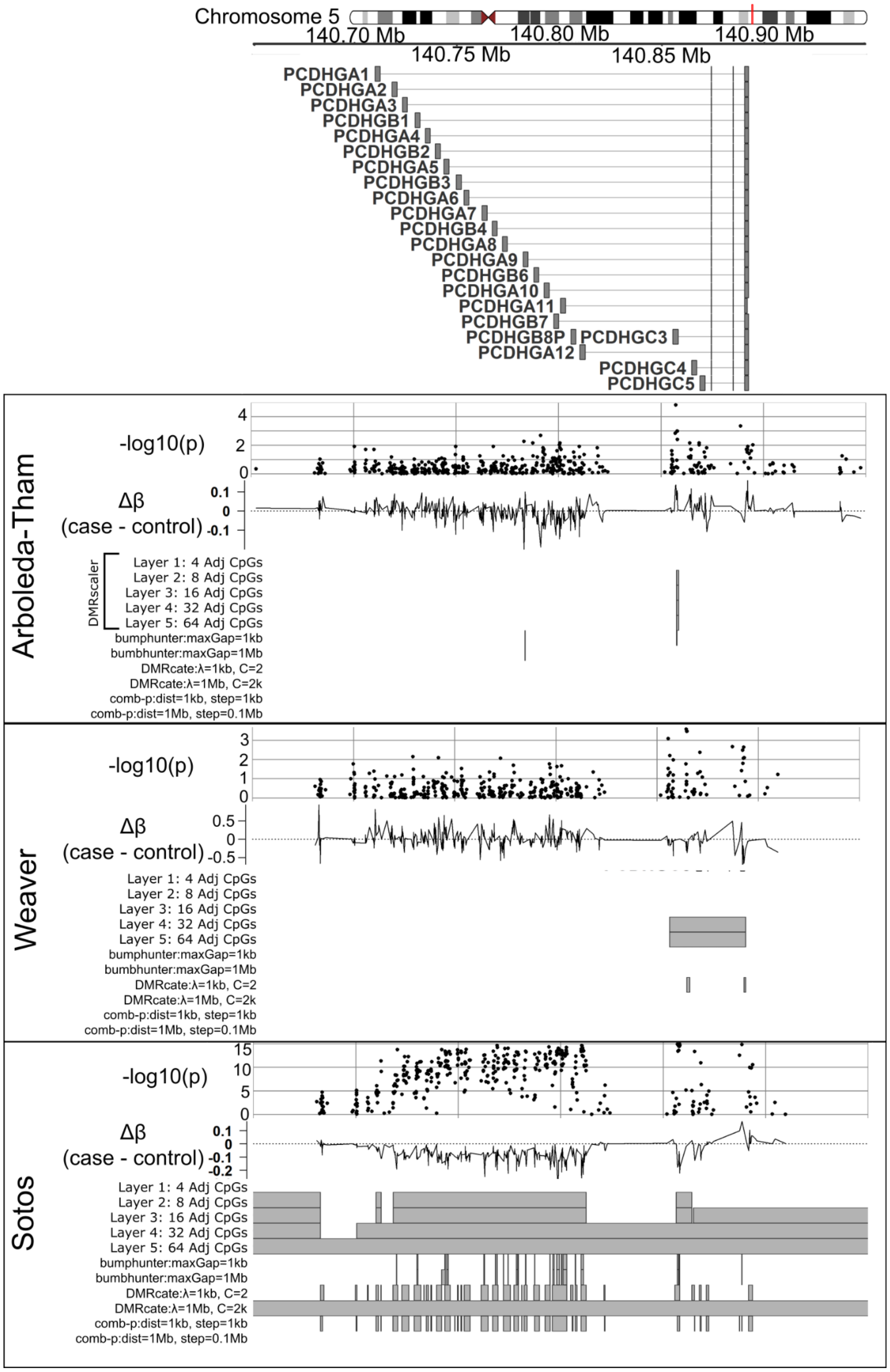

Figure S25 : *PCDHG* gene cluster GVIZ plot. PCDHG genes were identified as genes overlapped by some DMR in each of the syndrome datasets analyzed. Within each box, top track is -log10(p) from Wilcox test of beta values between cases and controls.Middle track shows -log10(p) significance value for the dataset specified at left. Next track shows Δβ, where $\Delta\beta = \beta_{Case} - \beta_{Control}$. Below the gene model track is the DMR track, highlighting the regions called as a DMR at each result layer from DMRscaler and from each competing method.

Arboleda-Tham samples	genomic position	coding change (NM_006766.3)	protein change (NP_006757.2)
Patient 1	chr8:g.41792353G>A	c.3385C>T	p.R1129*
Patient 2	chr8:g.41795056G>A	c.3070C>T	p.R1024*
Patient 3	chr8:g.41792353G>A	c.3385C>T	p.R1129*
Patient 4	chr8:g.41791630C>A	c.4108G>T	p.E1370*
Patient 5	chr8:g.41834753G>T	c.1136C>G	p.S379*
Patient 6	chr8:g.41794839_41794840insG	c.3286_3287insC	p.C1096Sfs*6
Patient 7	chr8:g.41791376dupC	c.4362dupG	p.T1455Dfs*9
Patient 8	chr8:g.41791085A>C	c.4653T>G	p.S1551R

Table S1: Arboleda-Tham Syndrome Patient Mutations

Syndrome Pair	Layer1 CGs in DMR	Layer2 CGs in DMR	Layer3 CGs in DMR	Layer4 CGs in DMR	Layer5 CGs in DMR
**Arboleda-Tham**	63	789	1472	3478	9231
**Sotos**	2671	52004	76386	177375	343144
**Weaver**	429	874	2101	4542	7797
**Arboleda-Tham: Sotos**	0	139	310	1351	6827
**Arboleda-Tham: Weaver**	0	14	18	143	424
**Sotos:Weaver**	38	333	601	2179	6308
**Arboleda-Tham: Sotos : Weaver**	0	5	25	93	469

Table S7: Raw Count of Measured CGs in DMRs called by *DMRscaler* : Layer1,2,3,4,5 are equivalent to 4,8,16,32,64 Adjacent CG Layers Respectively. CGs in DMR in each syndrome at each layer. Where multiple syndromes are listed, count represents CGs overlapped by some DMR in measured in each method using *DMRscaler*. Only the 425,733 Measured CGs present on both the Illumina 450k array, used for Sotos and Weaver, and the Illumina EPIC 850k array were used for overlap analysis.

Syndrome Pair	Layer1 OR (OR 95% CI)	Layer2 OR (OR 95% CI)	Layer3 OR (OR 95% CI)	Layer4 OR (OR 95% CI)	Layer5 OR (OR 95% CI)
**Arboleda-Tham : Sotos**	no overlap	1.72 (1.43-2.06) p=5.6e-8	1.37 (1.21-1.55) p=1.8e-6	1.04 (0.97-1.11) p=0.26	1.03 (0.98-1.08) p=0.20
**Arboleda-Tham :Weaver**	no overlap	9.94 (5.83-16.94) p=4.7e-10	2.8 (1.76-4.47) p=1.4e-4	4.58 (3.86-5.43) p=1.5e-46	3.00 (2.71-3.31) p=3.1e-77
**Sotos : Weaver**	17.16 (12.27-24.0) p=1.9e-32	4.95 (4.32-5.67) p=1.3e-95	2.06 (1.88-2.27) p=3.1e-45	1.52 (1.43-1.61) p=1.0e-43	1.55 (1.47-1.64) p=4.6e-56

Table S8: Odds Ratio (OR) for CGs found in DMR at each Layer of *DMRscaler* between all pairs of syndromes : Layer1,2,3,4,5 are equivalent to 4,8,16,32,64 Adjacent CG Layers respectively. Odds ratios (OR) are computed by labeling each measured CG as either in a DMR or not in a DMR for each syndrome to create a 2x2 contingency table to perform the odds ratio test on. Counts in Table S7. An confidence interval (CI) of the OR overlapping 1 implies no significant enrichment of CGs from one syndrome in the other.

Arboleda-Tham Analysis DMR Summary Table				
method	# DMRs	mean DMR width	median DMR width	% total width
dmrscaler				
4_loc_window_layer	14	20.41 kb	748 bp	0.0092%
8_loc_window_layer	151	73.92 kb	7.88 kb	0.36%
16_loc_window_layer	224	138.65 kb	34.66 kb	1.0%
32_loc_window_layer	293	261.62 kb	81.35 kb	2.5%
64_loc_window_layer	390	388.07 kb	144.59 kb	4.9%
bumphunter				
bumphunter_1	6443	161 bp	1 bp	0.034%
bumphunter_2	6674	2.93 kb	29 bp	0.63%
dmrcate				
dmrcate_1	45	771 bp	685 bp	0.0011%
dmrcate_2	30	1.22 kb	600 bp	0.0012%
combp				
combp_1	90	381 bp	344 bp	0.0011%
combp_3	263	599.10 kb	226.85 kb	5.1%

Table S10 : Summary of Arboleda-Tham analysis results

Weaver Analysis DMR Summary Table				
method	# DMRs	mean DMR width	median DMR width	% total width
**dmrscaler**				
4_loc_window_layer	83	1.10 kb	580 bp	0.0030%
8_loc_window_layer	123	1.55 kb	755 bp	0.0062%
16_loc_window_layer	182	4.67 kb	1.85 kb	0.028%
32_loc_window_layer	218	15.75 kb	4.28 kb	0.11%
64_loc_window_layer	226	54.64 kb	8.88 kb	0.40%
**bumphunter**				
bumphunter_1	58	177 bp	5 bp	0.00033%
bumphunter_2	55	359 bp	1 bp	0.00064%
**dmrcate**				
dmrcate_1	2560	1.10 kb	858 bp	0.092%
dmrcate_2	466	235.42 kb	1.71 kb	3.6%
**combp**				
combp_1	152	457 bp	374 bp	0.0023%
combp_3	396	416.17 kb	165.42 kb	5.4%

Table S11: Summary of Weaver analysis results

Sotos Analysis DMR Summary Table				
method	# DMRs	mean DMR width	median DMR width	% total width
dmrscaler				
4_loc_window_layer	507	1.52 kb	548 bp	0.025%
8_loc_window_layer	4295	38.12 kb	14.24 kb	5.3%
16_loc_window_layer	4754	69.20 kb	23.46 kb	11%
32_loc_window_layer	3845	258.23 kb	105.29 kb	32%
64_loc_window_layer	1776	1.22 Mb	555.13 kb	71%
bumphunter				
bumphunter_1	10336	155 bp	1 bp	0.052%
bumphunter_2	9819	1.11 kb	27 bp	0.36%
dmrcate				
dmrcate_1	26817	1.09 kb	903 bp	0.96%
dmrcate_2	282	8.37 Mb	5.26 Mb	77%
combp				
combp_1	34189	416 bp	202 bp	0.46%

Table S12 : Summary of Sotos analysis results
